# The efficacy and potential pharmacological mechanism of Fufang Danshen Tablet in promoting the rehabilitation of ischemic stroke: a meta-analysis and *in silico* study

**DOI:** 10.3389/fneur.2026.1724630

**Published:** 2026-02-12

**Authors:** Xuan Zeng, Yichu Nie, Jierong Mo, Zhangwen Peng, Tianen Zhou, Jun Jiang

**Affiliations:** 1 Department of Emergency, The First People's Hospital of Foshan (Foshan Hospital Affiliated to Southern University of Science and Technology), School of Medicine, Southern University of Science and Technology, Foshan, Guangdong, China; 2 Foshan Key Laboratory of Precision Therapy in Oncology and Neurology, Foshan, Guangdong, China; 3 State Key Laboratory of Neurology and Oncology Drug Development, Nanjing, Jiangsu, China; 4 Clinical Research Center, The First People's Hospital of Foshan (Foshan Hospital Affiliated to Southern University of Science and Technology), School of Medicine, Southern University of Science and Technology, Foshan, Guangdong, China; 5 Department of Medical Ultrasound, The First People's Hospital of Foshan (Foshan Hospital Affiliated to Southern University of Science and Technology), School of Medicine, Southern University of Science and Technology, Foshan, Guangdong, China

**Keywords:** efficacy, Fufang Danshen Tablet, *in silico* approach, ischemic stroke, meta-analysis, pharmacological mechanism

## Abstract

**Background:**

Fufang Danshen Tablet (FDT) is a traditional Chinese medicine (TCM) formula with remarkable efficacy in invigorating blood and eliminating blood stasis, thus commonly used in treating ischemic stroke. However, no systemic summary has been conducted to evaluate its efficacy yet. This study aims to determine the efficacy of FDT therapy for promoting the rehabilitation of ischemic stroke and to explore the potential pharmacological mechanism through an *in silico* approach.

**Methods:**

Eligible clinical trials involving FDT therapy for ischemic stroke were searched across nine online databases. Meta-analysis was conducted with RevMan and Stata software. Evaluation of the quality of evidence was performed on the GRADE system. Moreover, GEO datasets, network pharmacology, and molecular docking were employed to explore the potential pharmacological mechanism.

**Results:**

29 clinical trials concerning 1,634 participants were incorporated into the present meta-analysis. Compared with usual care alone, FDT combined with usual care exerted better efficacy in individuals with ischemic stroke, as evidenced by an elevated overall response rate and decreased National Institute of Health Stroke Scale (NIHSS), as well as the improvement of hemorheology, inflammation, and lipid metabolism. Moreover, meta-analysis of FDT individual intervention trials also showed significant therapeutic effects. Further network pharmacology and molecular docking analysis emphasized the potentially important role of the AKT/GSK3β/Cyclin D1 pathway for FDT to regulate oligodendrocyte precursor cells (OPCs) in treating ischemic stroke.

**Conclusion:**

FDT appears to significantly enhance neurological recovery, promote blood circulation, inhibit the inflammatory cascade, and lower blood lipid levels in patients with ischemic stroke. The AKT/GSK3β/Cyclin D1 pathway was predicted to be a potential mechanism of FDT in intervening ischemic stroke. In the future, more long-term follow-up RCTs with high quality are urgently needed, as well as experimental validations for the pharmacological mechanism.

**Systematic review registration:**

https://www.crd.york.ac.uk/PROSPERO/view/CRD420251015475, CRD420251015475.

## 1 Introduction

Stroke is a type of cerebrovascular disease characterized by a local blood supply disorder in the brain due to the infarction or rupture of cerebral vessels, leading to cerebral tissue ischemia, hypoxia, necrosis, and neurological deficit. At present, stroke has risen to be the leading cause of death in the Chinese population, with a fatality rate of about 343/100000, and the overall incidence of adults over 40 years old is about 500/100000 (1). Clinically, stroke mainly includes ischemic and hemorrhagic stroke, depending on the cause of the blood supply disorder. According to the latest data, ischemic stroke accounts for 69.6 to 72.8% of new strokes in the Chinese population (2). The fatality rate and complication rate of acute ischemic stroke patients in China during hospitalization were 0.5 and 12.8% (3). The fatality rate was 1.5 to 3.2% after 3 months and 3.4 to 6.0% after 1 year (4, 5). The disability rate was 14.6–23.1% after 3 months and 13.9–14.2% after 1 year (5, 6). The recurrence rate of stroke was 6.5% after 3 months and 10.3% after 1 year (5). Ischemic stroke has the epidemiological feature of high incidence, high fatality, high disability, and high recurrence, resulting in a serious family and social burden (7). Reperfusion is the primary goal of acute intervention in ischemic stroke. Although the advancement of intravenous thrombolysis and endovascular thrombectomy has significantly reduced the mortality rate of ischemic stroke, a large number of stroke patients still suffer from sequelae to varying degrees due to therapy-related risks (8). Chronic or permanent neurological dysfunction is the most common sequelae after ischemic stroke, which clearly impairs the patient’s quality of life (9, 10). For example, about 80% of patients have significant motor dysfunction at 3 months after stroke, and about 50% of patients continue to have motor dysfunction even 1 year later. In fact, about 20% of stroke patients even suffer long-term cognitive impairment, epilepsy, or dementia (11, 12). Hence, it is important to choose the appropriate medicament to cure ischemic stroke and its complications.

Traditional Chinese medicine (TCM) has distinct superiority in preventing and treating ischemic stroke (13–15). In the light of TCM theory, thrombosis is the primary cause of ischemic stroke; hence, invigorating blood and eliminating thrombosis is the key strategy to cure ischemic stroke. Salviae Miltiorrhizar Radix et Rhizoma, whose Chinese name is called Dan-Shen, is derived from the dried root and rhizome of *Salvia miltiorrhiza* Bge. It was first documented in the Shennong Bencao Jing and has been determined to be effective in improving blood circulation and eliminating thrombosis, making it a marvelous herb in treating cardiovascular and cerebrovascular diseases in China since the Eastern Han dynasty (A. D. 25–220) (16, 17). Notoginseng Radix et Rhizoma (San-Qi in Chinese), which is the dried root and rhizome of *Panax notoginseng* (Burk.) F. H., and initially documented in the Ben Cao Gang Mu (18), is another well-known herb for invigorating blood and eliminating thrombosis. In the 1970s, a modern TCM prescription named Fufang Danshen Tablet (FDT) was developed according to the clinical practices of Dan-Shen and San-Qi in treating cardiovascular and cerebrovascular diseases (19). It is made up of Dan-Shen, San-Qi, and Borneolum Syntheticum (Bing-Pian in Chinese) in the proportion of 450:141:8. Generally speaking, a TCM formula is combined according to the prescription regularity of Jun(emperor)–Chen(minister)–Zuo(adjuvant)–Shi(courier). As for FDT, Dan-Shen and San-Qi are assigned as Jun medicine and Chen medicine, respectively, playing a synergistic role in invigorating blood circulation and eliminating thrombosis (19). Bing-Pian is the Zuo and Shi medicine to regulate Qi and promote the delivery of effective constituents to the brain tissue. The combined use of these three TCM medicines can synergically exert the effect of improving blood circulation, eliminating thrombosis, regulating Qi, and alleviating pain, thus making FDT a frequently-used Chinese patent medicine in the intervention of ischemic stroke (20).

Since its launch in the 1970s, FDT has been involved in a great deal of clinical trials and experimental studies in treating ischemic stroke. However, there remain several gaps between scientific research and clinical application. On the one hand, no literature summary and evaluation have been conducted to determine its efficacy to date. On the other hand, the exact targets and pathways of the pharmacological mechanism underlying FDT’s treatment of ischemic stroke are still ambiguous. Here, we first carried out a comprehensive review and meta-analysis to define the effects of FDT for the intervention of ischemic stroke, such as improving cognitive impairment, hemorheology, inflammation, and lipid metabolism. In addition, we explore the potential mechanism of FDT in intervening ischemic stroke through GEO datasets, network pharmacology, and molecular docking, so as to provide a reference for further research and application of FDT.

## 2 Materials and methods

### 2.1 Meta-analysis to assess the efficacy of FDT against ischemic stroke

This systematic review adhered to the PRISMA statement (21). Moreover, the protocol has been registered in the PROSPERO database (CRD420251015475).

#### 2.1.1 Literature search strategy

The systemic literature search was performed by X. Zeng and Y. Nie independently, across the following nine databases covering literature from their inception to March 2025: Scopus, PubMed, Embase, Web of Science, the Cochrane Library, the Chinese National Knowledge Infrastructure, the Chinese Biomedical Database, the WanFang Database, and the Chinese Science and Technology Journals Database. There are no restrictions on the level and language of the article. Relevant keywords used in the literature search include ischemic stroke, cerebral infarction, brain infarction, Fufang Danshen Tablet, Compound Danshen Tablet, and so on. The reference lists were further examined manually to determine the availability of retrieved articles and any potentially undiscovered relevant research.

#### 2.1.2 Inclusion criteria

(a) Study type: Clinical trials evaluating the efficacy of FDT against ischemic stroke.
(b) Participants: All included participants were consistent with the diagnosis of ischemic stroke, regardless of age, sex, race, nationality, or disease severity.
(c) Interventions: The intervention was oral FDT therapy with or without usual care (UC), regardless of dose and duration of treatment. UC refers to the necessary treatment for ischemic stroke, especially during the acute phase, such as antiplatelet aggregation, brain protection, and plaque stabilization therapy, etc.
(d) Comparators: To comprehensively determine the efficacy of FDT in treating ischemic stroke, this study included patients treated with FDT combined or without UC, so as to carry out subgroup analysis. Based on the data reported in the literature, the intervention group of FDT-combined UC was compared with the UC control group, that is, randomized controlled trials (RCTs). Unfortunately, owing to the absence of a control group in certain studies, the therapeutic effect of FDT alone was evaluated by comparing the level of disease indicators before and after treatment, regarded as single-arm trials (SATs).
(e) Outcome measures: The primary outcomes were overall response rates and the National Institute of Health Stroke Scale (NIHSS). The overall response rate was calculated in light of the Scoring Criteria of Clinical Neurological Deficit in Stroke Patients. Briefly, the decrease of neurological deficit score > 90, 45–90%, 18–45%, and <18% were considered as basically cured, significant progress, progress, and ineffective, respectively. Basically, cure and significant progress cases were included in the calculation of the overall response rate. The secondary outcomes were hemorheology indexes, inflammatory markers, and blood lipid indicators, including whole blood viscosity at high shear rate (WBV-h), whole blood viscosity at low shear rate (WBV-l), plasma viscosity, hematocrit (HCT), interleukin-6 (IL-6), tumor necrosis factor-*α* (TNF-α), hypersensitive C-reactive protein (hs-CRP), total cholesterol (TC), triglyceride (TG), low density lipoprotein cholesterol (LDL-c), and high density lipoprotein cholesterol (HDL-c).

#### 2.1.3 Exclusion criteria

(a) Reviews, animal studies, conference reports, or case reports.
(b) Duplicate publications.
(c) The articles did not report outcomes relevant to this study.
(d) Full text or relevant data were not available.

#### 2.1.4 Data extraction

In the light of inclusion and exclusion criteria, trial selection and data extraction were independently conducted and then cross-checked by two investigators (X. Zeng and Y. Nie). Any different opinions were addressed with discussion or third-party adjudication. The records searched from nine databases were imported into NoteExpress 4.1.0 and screened by inspecting the title and abstract of the articles after removing repeated publications, non-clinical studies, and reviews. Subsequently, the full text of the potentially conforming literature was read to define the eligibility. Relevant data were organized in Microsoft Excel, including: (a) basic information of the literature, including title, author, and publication year; (b) patient features, such as gender, age, sample size, and course of disease; (c) intervention and control information, including dosage and duration of treatment; (d) outcome measures; and (e) factors used to assess the risk of bias.

#### 2.1.5 Quality assessment

The quality of involved clinical trials was evaluated using the Cochrane bias risk assessment tool (22). X. Zeng and Y. Nie independently reviewed and assessed the basis of six categories so as to determine the level of potential bias. Any dissension was settled by detailed discussion and checked by an independent investigator (T. Zhou).

#### 2.1.6 Data synthesis

Data synthesis was carried out separately for RCTs of FDT combined intervention and SATs treated with FDT alone, and performed on ReviewManager 5.3.5 software. For continuous variables, the weighted standardized mean difference (SMD) or mean difference (MD) and its 95% confidence interval (CI) were used as the effect indicators, while the risk ratio (RR) and its 95%CI were selected for binary variables. *p* < 0.05 was considered a statistically significant difference. Cochrane’s Q test combined I^2^ value was employed to assess the heterogeneity, and the significance level of the heterogeneity test is *p* < 0.05. If *p* > 0.05 and I^2^ ≤ 50%, the level of heterogeneity is low, and the fixed effect model was selected to pool the effect size. On the contrary, *p* ≤ 0.05 or I^2^ > 50% indicated that the inter-study heterogeneity is at a high level, leading to the selection of the random effects model. Sensitivity analysis was conducted by excluding individual trials to determine their influence on the overall results. If the number of studies involving a certain outcome indicator was more than 5, the funnel plot and Egger’s test were employed to determine the publication bias.

#### 2.1.7 Quality of evidence

With the GRADE online tool named GRADEpro GDT, the quality of evidence for outcome measures was evaluated as very low, low, medium, or high in the light of given evaluation items (23).

### 2.2 *In silico* approach to explore the potential pharmacological mechanism of FDT in treating ischemic stroke

#### 2.2.1 GEO datasets analysis

Oligodendrocytes (OLs) are indispensable myelin architects in the central nervous system and profoundly influence the functions of neuronal axons (24). After the onset of ischemic stroke, OLs suffer from oxygen and nutrient deprivation, resulting in demyelination, axonal degeneration, and even neuronal death, thereby causing neurological dysfunction (9). The differentiation of oligodendrocyte precursor cells (OPCs) into mature OLs plays a fundamental role in remyelination, and regulating OPCs to promote remyelination has become a hot spot for improving neurological dysfunction (25, 26).

However, the effects of FDT and its components on OPCs and related remyelination remain unclear. Herein, we first adopt the Gene Expression Omnibus (GEO) database (https://www.ncbi.nlm.nih.gov/geo/) combined with bioinformatics analysis to explore the target genes of OPCs for remyelination. In the GEO database, the relevant datasets were retrieved and extracted with the keywords “stroke” and “oligodendrocyte precursor cells”. As a result, the high-throughput sequencing datasets GSE53737 and GSE114609 were obtained. GSE53737 investigated the effects of ischemic stroke on OPCs in mice using RNA sequencing technology, including 15 samples of ischemic stroke models and 10 normal control samples. GSE114609 evaluated the effect of oxygen–glucose deprivation on OPCs cultured *in vitro*, including four OGD model samples and four normal control samples. The differentially expressed genes (DEGs) were screened using DESeq2 software, and *p* < 0.05, |log2(FC)| ≥ 1.5 was used as the screening criterion for DEGs.

#### 2.2.2 Network pharmacology analysis

The chemical components absorbed into the body are the material basis for traditional Chinese medicine to exert its pharmacological effects. In a previous study, we identified 30 components from the rat serum samples after oral administration of FDT, using a high-resolution UHPLC-Q-TOF-MS/MS technology (27). Based on the above results, we input these compounds into the PubChem database (https://pubchem.ncbi.nlm.nih.gov/) to acquire the corresponding SMILES numbers (Detailed in Supplementary Table S1). Subsequently, we introduced these SMILES numbers into the input Swiss Target Prediction database (http://swisstargetprediction.ch/), so as to obtain the potential targets of the mentioned 30 components absorbed into the blood.

The potential targets of 30 components were intersected with the above-mentioned DEGs to obtain drug-disease common target genes, which were subsequently introduced into the STRING database (https://string-db.org/) to obtain the protein–protein interaction (PPI) network. Then, visual analysis was conducted in the Cytoscape software, and the topological parameters of the relevant targets were analyzed using the CytoNCA plugin. The importance of the nodes was evaluated based on the node Degree value (Degree) and betweenness centrality (BC) to screen out the key targets. Further GO analysis and KEGG pathway enrichment were performed on Database for Annotation, Visualization, and Integrated Discovery (DAVID) (https://davidbioinformatics.nih.gov/). Subsequently, the obtained enrichment analysis results were visualized through online tools (https://bioinformatics.com.cn/), and the bubble chart was drawn.

#### 2.2.3 Molecular docking

The crystal structures of key target proteins were acquired from the PDB database (http://www.rcsb.org/), and the structural files of 30 active ingredients were obtained from the PubChem database. Molecular docking analysis was carried out using AutoDockVina software. Visualization processing was performed using PyMol and DiscoveryStudio software.

## 3 Results

### 3.1 Meta-analysis results

#### 3.1.1 Retrieval of eligible studies

**Figure 1:**
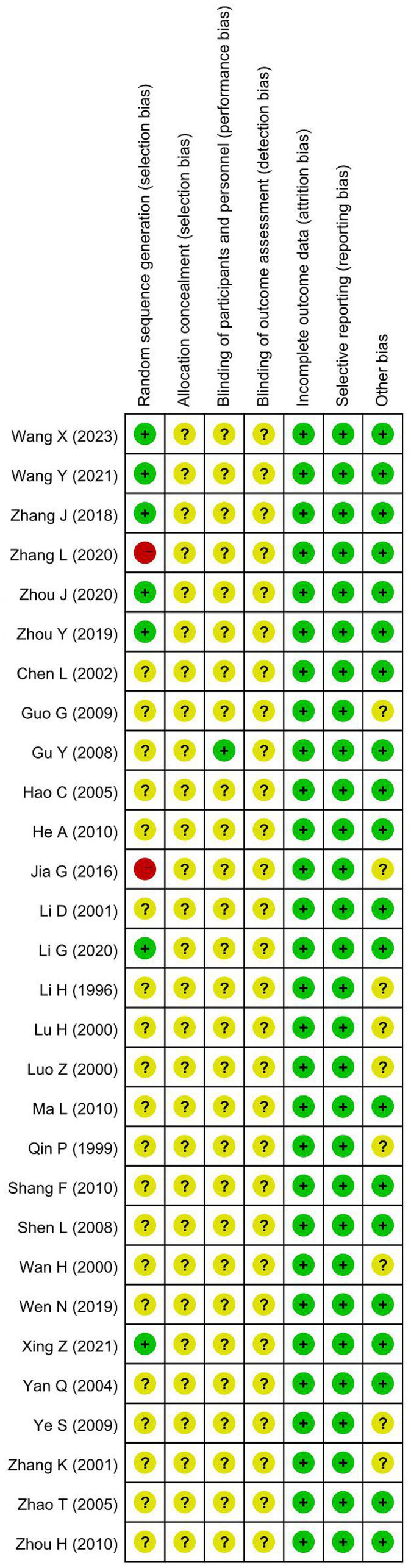
Flowchart of the study selection process.

According to the retrieval scheme, a total of 1,216 literature were initially searched and 692 were retained after removing duplicate publications. After reading the titles and abstracts, 589 articles, including conference papers, case reports, meta-analyses, reviews, *in vitro* and animal experiments, therapeutic drug discrepancies, and other unrelated literature, were excluded. The full text of remanent 103 articles was carefully evaluated for eligibility. A total of 74 studies were discarded because of the following factors: repeated publication, incomplete data, unavailable full-text, inconsistent diseases, or interventions. Finally, 29 studies were selected for meta-analysis, including 6 RCTs of FDT-combined intervention and 23 SATs of FDT-individual intervention. The process of study selection was charted in Figure 1.

#### 3.1.2 Characteristics of included trials

As shown in Table 1, the included trials were carried out in China and reported between 1996 and 2023. A total of 1,634 patients were involved, with the age range from 36 to 93. The time from onset to enrollment of participants in different trials varied greatly, ranging from 6 h to 20 years. The dosage of FDT intervention was mostly three tablets each time and three times per day. The course of treatment lasted between 7 days and 24 weeks, with a typical treatment period of 1 month (28 ~ 30 days). As for outcome measures, 6 trials reported overall response rate, 10 trials reported NIHSS score, 20 trials reported the level of WBV-h and plasma viscosity, 19 trials reported WBV-l level, 18 trials reported HCT level, 2 trials reported the level of IL-6, TNF-*α*, and hs-CRP, 14 trials reported the level of TG and TC, 3 trials reported LDL-c level, and 10 trials reported HDL-c level.

**Table 1:** Characteristics of included trials.

First author (public year)	Sample size	Gender (M/F)	Age (year)	Time from onset to enrollment (Mean)	Interventions^a^	Duration of treatment	Outcomes^b^
Controlled clinical trial treated with FDT-combined usual care (UC) (FDT-combined intervention)							
Wang X (2023) (31)	T: 40 C: 40	43/37	T: 58.35 ± 1.61 C: 58.12 ± 1.65	T: 11.52 ± 0.61 h C: 11.69 ± 0.58 h	T: FDT + UC (0.78 g/time, tid, p.o.) C: UC	7 d	①②③⑤⑥⑦⑧
Wang Y (2021) (32)	T: 41 C: 41	50/32	T: 43–79 (64.15 ± 7.51) C: 42–77 (64.18 ± 7.64)	T: 9–30 h (25.61 ± 2.3 h) C: 10–30 h (25.7 ± 2.12 h)	T: FDT + UC (0.75 g/time, tid, p.o.) C: UC	15 d	①②⑦⑧⑨
Zhang J (2018) (34)	T: 30 C: 30	32/28	T: 44–68 (53.87 ± 7.80) C: 46–67 (54.17 ± 7.26)	T: 6 d–17 w (8.66 ± 2.34 w) C: 1–18 w (8.72 ± 2.36 w)	T: FDT + UC (0.96 g/time, tid, p.o.) C: UC	24 w	①②③④⑤⑥⑩⑪⑫⑬
Zhang L (2020) (28)	T: 82 C: 82	59/35	T: 46–70 (55.7 ± 4.7) C: 47–71 (56.1 ± 4.3)	T: 9 d–18 w (9.7 ± 3.6 w) C: 1–17 w (9.1 ± 3.8 w)	T: FDT + UC (0.96 g/time, tid, p.o.) C: UC	24 w	①②③④⑤⑥⑩⑪⑫⑬
Zhou J (2020) (35)	T: 36 C: 36	41/31	T: 40–75 C: 40–75	T: ≤72 h C: ≤72 h	T: FDT + UC (0.96 g/time, tid, p.o.) C: UC	14 d	①②⑩⑪⑫⑬
Zhou Y (2019) (36)	T: 40 C: 40	46/34	T: 44–77 (64.84 ± 8.64) C: 46–75 (64.58 ± 8.72)	T: 9–26 h (13.28 ± 1.62 h) C: 10–28 h (13.58 ± 1.59 h)	T: FDT + UC (0.96 g/time, tid, p.o.) C: UC	14 d	①②③④⑤⑥⑨
Clinical trials treat with FDT alone (FDT individual intervention)							
Chen L (2002) (68)	T: 40	58/34	40–69 (66.43 ± 7.09)	T: 36.05 ± 37.46 d	T: FDT (4 tablets/time, tid, p.o.)	30 d	③④⑤⑥
Gu Y (2008) (37)	T: 50	32/18	T: 50–80 (67)	T: 7–20 m (13.5 m)	T: FDT (3 tablets/time, tid, p.o.)	28 d	②
Guo G (2009) (69)	T: 23	NR	T: 36–82 (60.6 ± 2.3)	T: 6 h–3 d	T: FDT (3 tablets/time, tid, p.o.)	28 d	③④⑤⑥
Hao C (2005) (70)	T: 35	20/15	T: 44–79 (61.7)	NR^c^	T: FDT (0.96 g/time, tid, p.o.)	14 d	③④⑤⑥
He A (2010) (50)	T: 68	39/29	T: 59 ± 15	NR	T: FDT (0.96 g/time, tid, p.o.)	21 d	③⑩⑪⑬
Jia G (2016) (29)	T: 114	NR	T: 43–77 (61.2 ± 5.3)	NR	T: FDT (3 tablets/time, tid, p.o.)	28 d	②
Li D (2001) (51)	T: 30	20/10	T: 44–79 (64)	T: 21–62 d	T: FDT (3 tablets/time, tid, p.o.)	28 d	③④⑤⑩⑪⑬
Li G (2020) (30)	T: 42	25/17	T: 41–84 (58.4 ± 4.3)	NR	T: FDT (0.96 g/time, tid, p.o.)	14 d	②
Li H (1996) (71)	T: 61	41/20	T: 45–79 (59 ± 7)	T: 8–72 h (21 ± 5 h)	T: FDT (3 tablets/time, tid, p.o.)	30 d	③⑤⑥
Lu H (2000) (72)	T: 20	NR	T: 45–76 (62)	T: 6 m–16 y	T: FDT (0.96 g/time, tid, p.o.)	30 d	④⑥
Luo Z (2000) (73)	T: 64	37/27	T: 50–70 (59.4)	NR	T: FDT (3 tablets/time, tid, p.o.)	30 d	④
Ma L (2010) (59)	T: 30	15/15	T: 36–75 (52.12 ± 0.19)	NR	T: FDT (0.96 g/time, tid, p.o.)	30 d	⑩⑪⑬
Qin P (1999) (52)	T: 60	40/20	T: 44–79 (64)	T: 21–62 d	T: FDT (3 tablets/time, tid, p.o.)	28 d	③④⑤⑩⑪⑬
Shang F (2010) (53)	T: 30	18/12	T: 40–76 (57.2 ± 9.04)	NR	T: FDT (3 tablets/time, tid, p.o.)	30 d	③④⑤⑥⑩⑪
Shen L (2008) (54)	T: 28	18/10	T: 61.2 ± 4.5	T: 3.0 ± 2.4 m	T: FDT (3 tablets/time, tid, p.o.)	28 d	③④⑤⑩⑪
Wan H (2000) (60)	T: 38	28/10	T: 40–75 (62)	T: 1–35 m (11 m)	T: FDT (not report dosage)	28 d	⑩⑪⑬
Wen N (2019) (55)	T: 50	23/27	T: 45–82 (58.46 ± 3.59)	T: 0.5–3 y (2.03 ± 0.12 y)	T: FDT (0.96 g/time, tid, p.o.)	30 d	③④⑤⑥⑩⑪
Xing Z (2021) (33)	T: 40	22/18	T: 48–69 (53.12 ± 7.12)	T: 1–17 w (8.77 ± 2.21 w)	T: FDT (0.96 g/time, tid, p.o.)	28 d	②③④⑤⑥
Yan Q (2004) (56)	T: 40	NR	T: 48–93 (72)	NR	T: FDT (4 tablets/time, tid, p.o.)	30 d	③④⑤⑥⑩⑪
Ye S (2009) (74)	T: 47	34/13	T: 53–81 (67.05 ± 10)	NR	T: FDT (0.90 g/time, tid, p.o.)	28 d	③④⑤⑥
Zhang K (2001) (57)	T: 83	NR	NR	NR	T: FDT (1.28 g/time, tid, p.o.)	20 d	③④⑤⑥⑩⑪⑬
Zhao T (2005) (58)	T: 63	NR	NR	NR	T: FDT (1.28 g/time, tid, p.o.)	20 d	③④⑤⑥⑩⑪⑬
Zhou H (2010) (75)	T: 40	23/17	T: 42–78 (63.6 ± 7.56)	NR	T: FDT (3 tablets/time, tid, p.o.)	28 d	③④⑤⑥

^a^Some studies did not describe the weight specifications of FDT, so only the number of tablets was listed. For commercially available FDT, its specification is generally 0.32 g per tablet, which is equivalent to 0.6 g of TCM decoction pieces.

^b^①: overall response rate, ②: National Institute of Health Stroke Scale (NIHSS), ③: whole blood viscosity at high shear rate (WBV-h), ④: whole blood viscosity at low shear rate (WBV-l), ⑤: plasma viscosity, ⑥: hematocrit (HCT), ⑦: interleukin-6 (IL-6), ⑧: tumor necrosis factor-α (TNF-α), ⑨: hypersensitive C-reactive protein (hs-CRP), ⑩: triglyceride (TG), ⑪: total cholesterol (TC), ⑫: low-density lipoprotein cholesterol (LDL-c), ⑬: high-density lipoprotein cholesterol (HDL-c).

^c^NR, not reported.

#### 3.1.3 Risk of bias assessment

**Figure 2:**
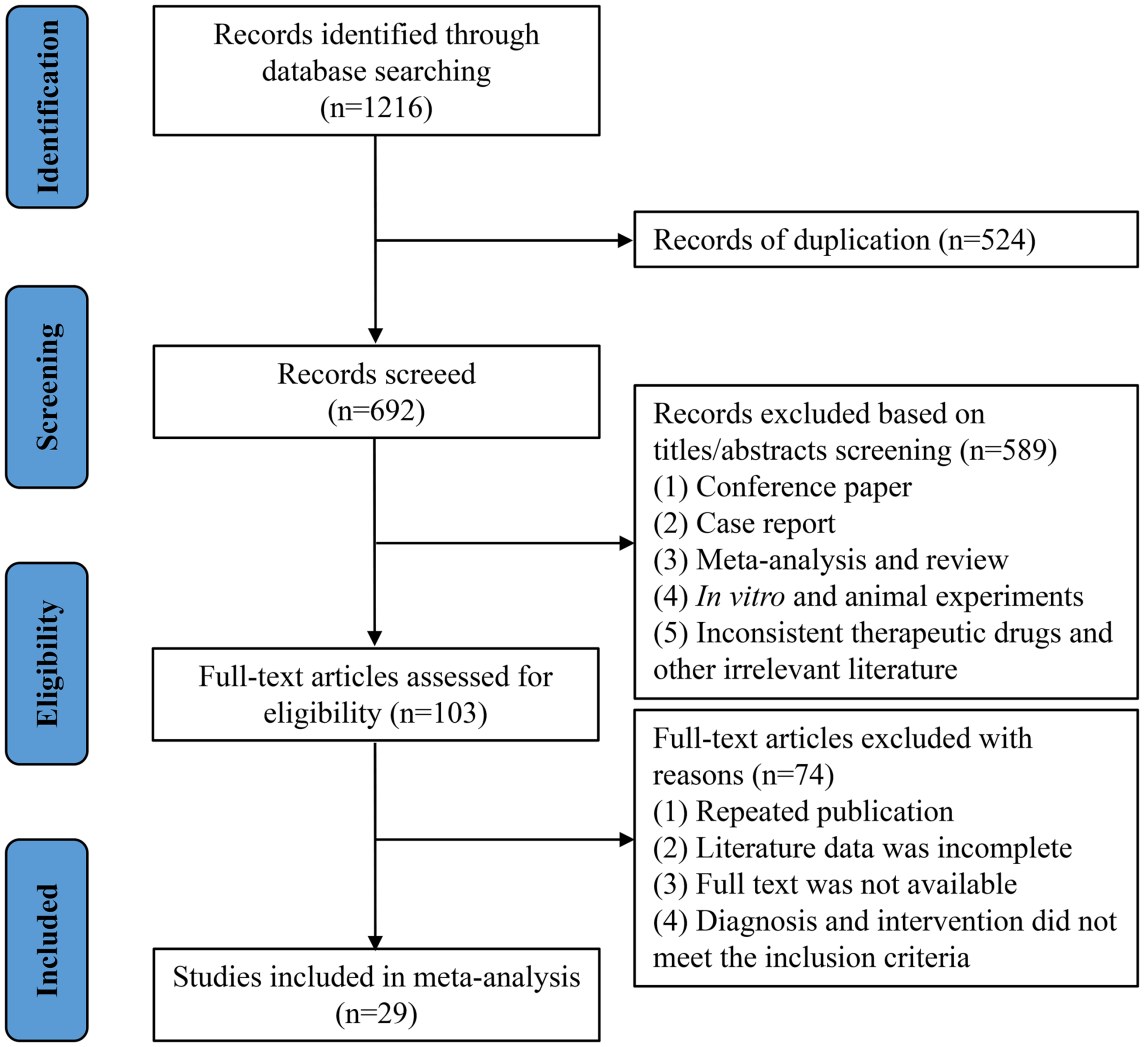
Risk of bias graph for included studies.

**Figure 3:**
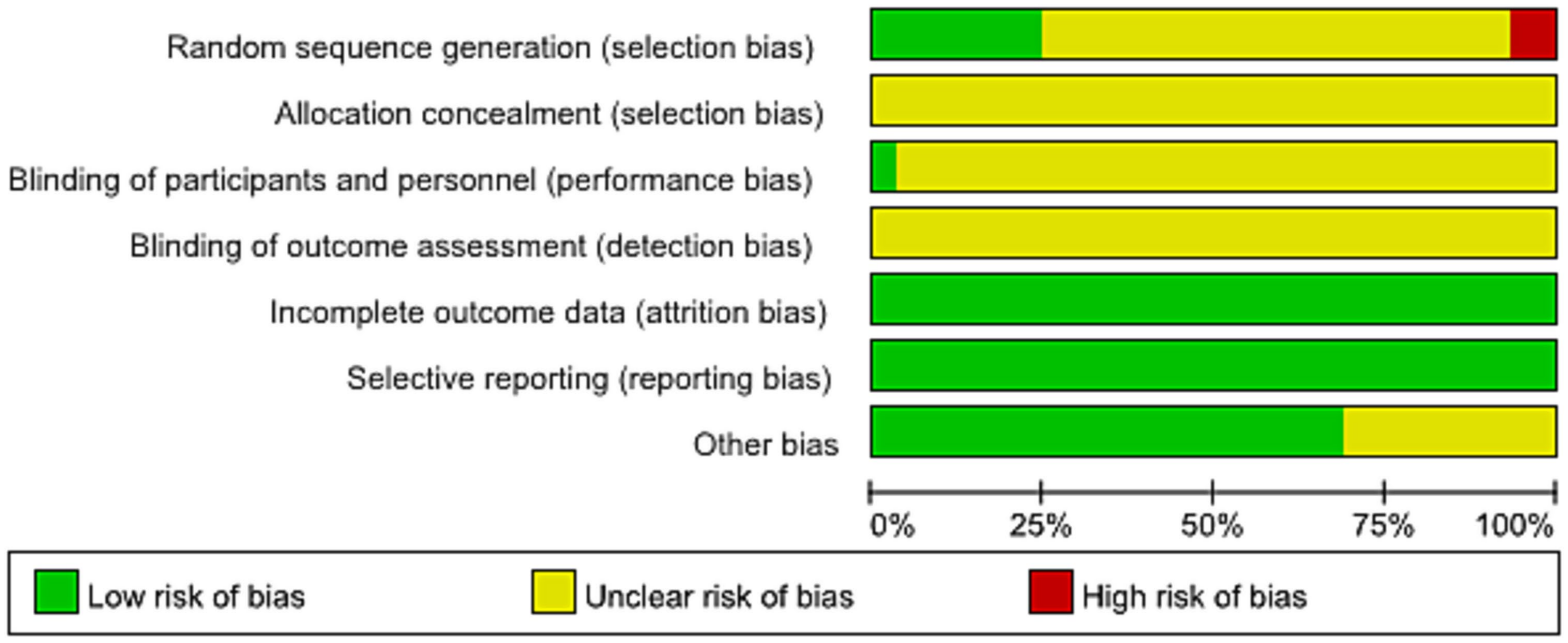
Risk of bias summary for included studies.

The quality of the 29 included trials was assessed in light of the Cochrane Handbook for Systematic Reviews of Interventions, and corresponding results are presented in Figures 2, 3. Two works were rated to be high-risk because the participants were grouped according to treatment method (28) or treatment number (29). Seven studies (30–36) using random number tables were judged to be low-risk, and the remaining twenty studies only reported random grouping without specifying the method and were evaluated as unclear. Since none of the trials specifically depict the procedure of allocation concealment, they were evaluated as unclear. A study (37) reported a double-blind design and was assigned to be low-risk, and the other trials were not specified and were rated as unclear. None of the studies specifically described the blind situation of outcome assessment, which was evaluated as unclear. All studies reported pre-specified outcome measures and were evaluated as low-risk. Other sources of bias mainly consider the balance of baseline data between the intervention and the control group, as well as whether the intervention protocol, follow-up process, and data collection method were clearly described.

#### 3.1.4 Outcome measures

**Figure 4:**
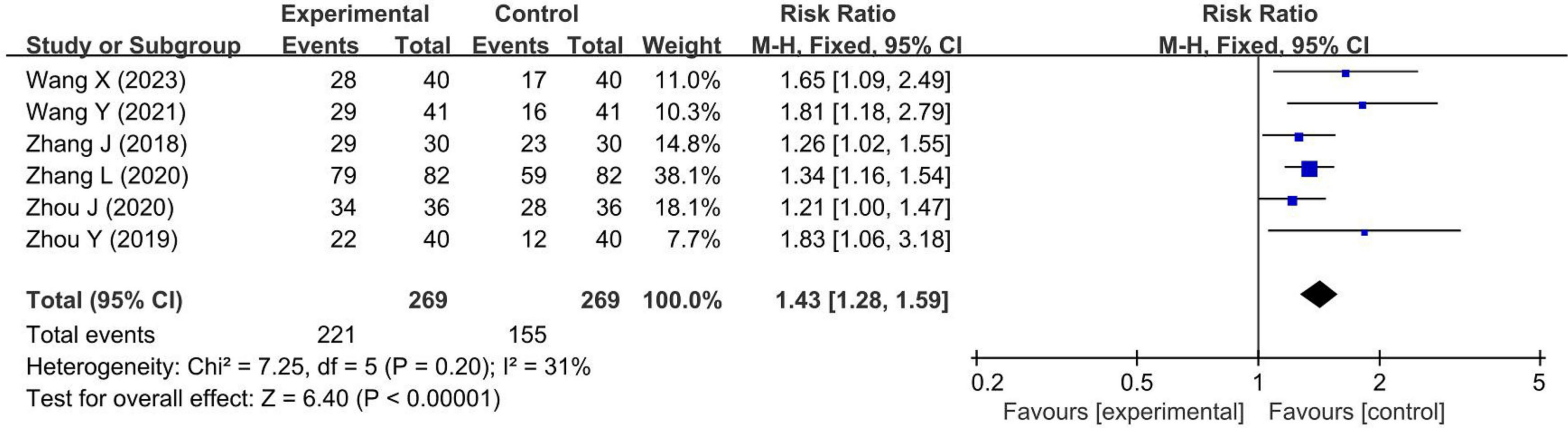
Forest plot for the meta-analysis of the overall response rate.

**Figure 5:**
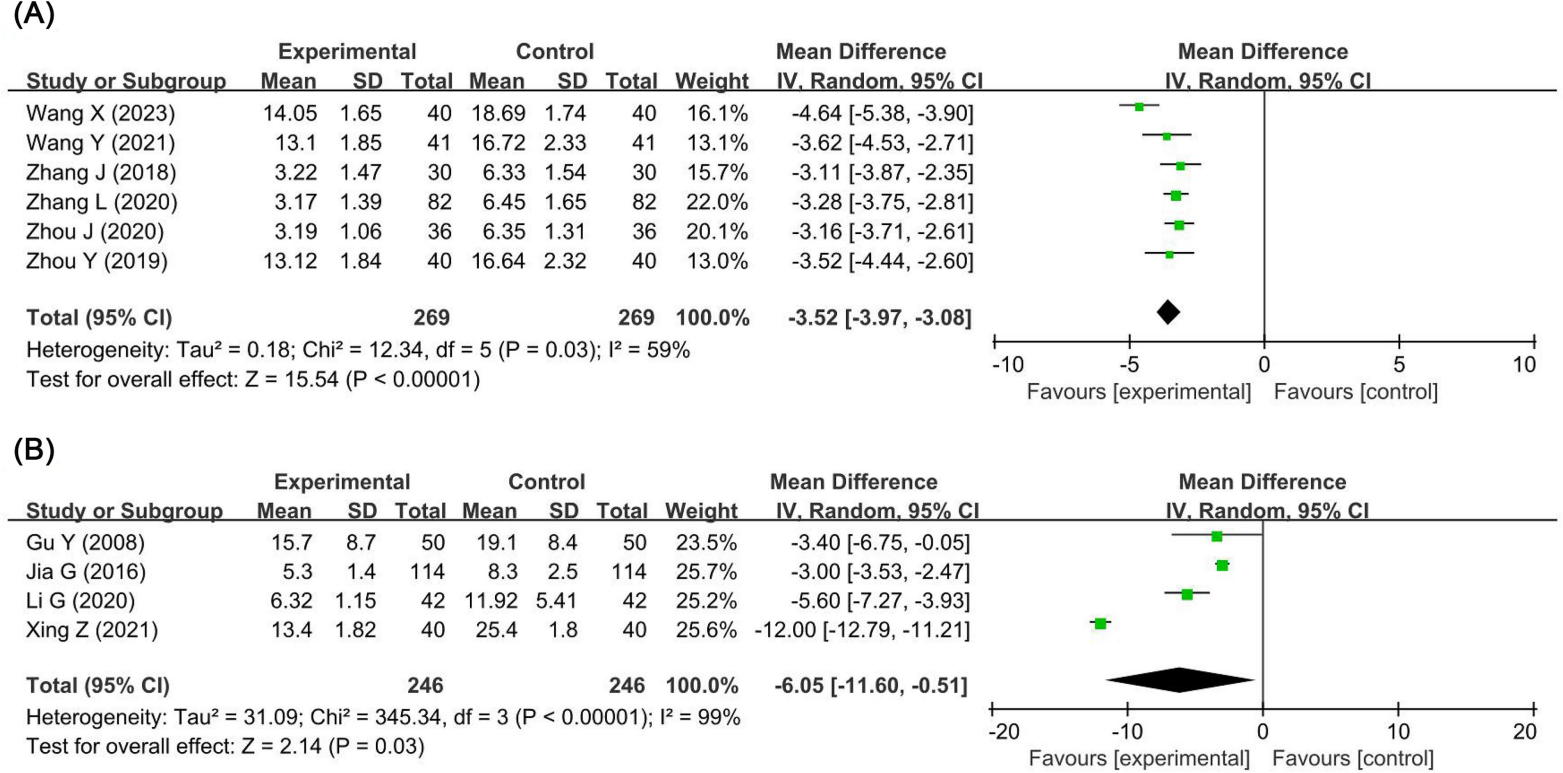
Forest plot for the meta-analysis of NIHSS score. **(A)** RCTs of the FDT-combined intervention. **(B)** SATs of FDT combined intervention.

Results of data synthesis for RCTs of FDT-combined intervention and SATs of FDT-individual intervention are summarized in Table 2. Overall response rate, NIHSS score, and 11 other secondary outcomes were involved. Based on included RCTs, the FDT-combined intervention exerted superior efficacy than usual care alone in ischemic stroke patients, which was evidenced by elevated overall response rate (Figure 4) and decreased NIHSS score (Figure 5), as well as reduced hemorheology indexes, inflammatory markers, and blood–lipid indicators (Supplementary Figures S1–S9). What’s more, the SATs of FDT-individual intervention also revealed the significant therapeutic effects in reducing NIHSS score, blood viscosity, and lipid level.

**Table 2:** Summary of meta-analysis results.

Outcomes	No. of studies	No. of patients	OR or MD (95%CI)	*p* value	Heterogeneity	
					Q (*p* value)	I^2^ (%)
RCTs of FDT combined intervention						
Overall response rate	6 (27, 30, 31, 33–35)	538	1.43 [1.28, 1.59]	<0.00001	0.20	31
NIHSS score	6 (27, 30, 31, 33–35)	538	-3.52 [−3.97, −3.08]	<0.00001	0.03	59
WBV-h	4 (27, 30, 33, 35)	384	−2.09 [−3.53, −0.66]	0.004	<0.00001	99
WBV-l	3 (27, 33, 35)	304	−2.96 [−4.92, −0.99]	0.003	<0.00001	98
Plasma viscosity	4 (27, 30, 33, 35)	384	−0.66 [−1.25, −0.08]	0.03	<0.00001	99
HCT	4 (27, 30, 33, 35)	384	−6.03 [−8.42, −3.64]	<0.00001	<0.00001	97
IL-6	2 (30, 31)	162	−2.55 [−4.40, −0.69]	0.007	0.006	87
TNF-α	2 (30, 31)	162	−4.47 [−5.17, −3.77]	<0.00001	0.17	46
hs-CRP	2 (30, 35)	160	−1.57 [−1.68, −1.46]	<0.00001	0.55	0
TG	3 (27, 33, 34)	296	−0.16 [−0.25, −0.06]	0.001	0.04	69
TC	3 (27, 33, 34)	296	−0.72 [−0.81, −0.63]	<0.00001	1.00	0
LDL-c	3 (27, 33, 34)	296	−0.20 [−0.32, −0.08]	0.001	0.08	61
HDL-c	3 (27, 33, 34)	296	0.11 [0.03, 0.18]	0.005	0.09	58
SATs of FDT individual intervention						
NIHSS score	4 (28, 29, 32, 36)	246	−6.05 [−11.60, −0.51]	0.03	<0.00001	99
WBV-h	16 (32, 37, 50–58, 68–71, 74)	738	−0.45 [−0.61, −0.25]	<0.00001	<0.00001	81
WBV-l	16 (32, 37, 50, 52–58, 68, 69, 71, 72, 74, 75)	693	−1.16 [−1.53, −0.79]	<0.00001	<0.00001	87
Plasma viscosity	16 (32, 37, 50–58, 68, 69, 71, 73, 74)	670	−0.14 [−0.22, −0.06]	0.0006	<0.00001	89
HCT	14 (32, 37, 51, 52, 54–58, 68, 69, 73–75)	572	−0.73 [−1.15, −0.30]	0.0008	<0.00001	92
TG	11 (50, 52–54, 56, 57, 59, 70, 71, 73, 74)	520	−0.45 [−0.61, −0.30]	<0.00001	<0.00001	83
TC	11 (50, 52–54, 56, 57, 59, 70, 71, 73, 74)	520	−0.50 [−0.70, −0.30]	<0.00001	0.0002	70
HDL-c	7 (50, 56, 57, 59, 70, 71, 73)	372	0.32 [0.16, 0.49]	<0.0001	<0.00001	96

#### 3.1.5 Publication bias

**Figure 6:**
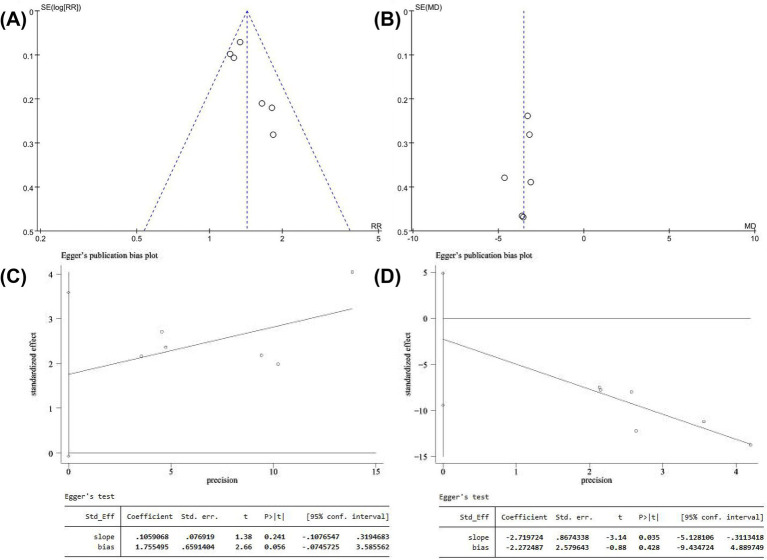
The funnel plot and Egger’s test results of overall response rate **(A,C)** and NIHSS score **(B,D)**.

As shown in Table 1, there were six FDT combined intervention trials reported overall response rate and NIHSS scores. There is a mild publication bias that was detected in the funnel plot of the overall response rate and NIHSS, but Egger’s test showed that there is no publication bias of statistical significance (Figure 6). For FDT-individual intervention, more than five studies reported outcomes of WBV-h, WBV-l, HCT, TG, TC, and HDL-c. A slight publication bias was observed in the funnel plot of most outcomes, while Egger’s test indicated no publication bias of statistical significance, except for WBV-l and TC (Supplementary Figures S10–S16).

#### 3.1.6 Quality of evidence

The GRADE online tool, named GRADEpro GDT, was employed in evaluating the quality of evidence for thirteen outcomes. For RCTs of FDT combined intervention, overall response rate, TNF-*α*, hs-CRP, and TC were rated as moderate. NIHSS, TG, LDL-c, and HDL-c were rated as low, while WBV-h, WBV-l, plasma viscosity, HCT, and IL-6 were rated as very low (Table 3). As for the SATs of FDT individual intervention, all outcomes were rated as very-low-quality evidence due to a non-randomized design (Supplementary Table S1).

**Table 3:** GRADE evidence profiles.

Outcomes	Certainty assessment							No. of patients		Effect		Certainty	Importance
	No. of studies	Study design	Risk of bias	Inconsistency	Indirectness	Imprecision	Other considerations	Treatment with FDT combined usual care	Treatment with usual care	Relative (95% CI)	Absolute (95% CI)		
Overall response rate	6	Randomized trials	Serious	Not serious	Not serious	Not serious	None	221/269 (82.2%)	155/269 (57.6%)	RR 1.43 (1.28 to 1.59)	248 more per 1,000 (from 161 more to 340 more)	Moderate	CRITICAL
NIHSS	6	Randomized trials	Serious	Serious^a^	Not serious	Not serious	None	269	269	–	MD 3.52 lower (3.97 lower to 3.08 lower)	Low^a^	CRITICAL
WBV-h	4	Randomized trials	Serious	Very serious^b^	Not serious	Not serious	None	192	192	–	MD 2.09 lower (3.53 lower to 0.66 lower)	Very low^b^	IMPORTANT
WBV-l	3	Randomized trials	Serious	Very serious^b^	Not serious	Not serious	None	152	152	–	MD 2.96 lower (4.92 lower to 0.99 lower)	Very low^b^	IMPORTANT
Plasma viscosity	4	Randomized trials	Serious	Very serious^b^	Not serious	Not serious	None	192	192	–	MD 0.66 lower (1.25 lower to 0.08 lower)	Very low^b^	IMPORTANT
HCT	4	Randomized trials	Serious	Very serious^b^	Not serious	Not serious	None	192	192	–	MD 6.03 lower (8.42 lower to 3.64 lower)	Very low^b^	IMPORTANT
IL-6	2	Randomized trials	Serious	Very serious^b^	Not serious	Not serious	None	81	81	–	MD 2.55 lower (4.4 lower to 0.69 lower)	Very low^b^	IMPORTANT
TNF-α	2	Randomized trials	Serious	Not serious	Not serious	Not serious	None	81	81	–	MD 4.47 lower (5.17 lower to 3.77 lower)	Moderate	IMPORTANT
hs-CRP	2	Randomized trials	Serious	Not serious	Not serious	Not serious	None	81	81	–	MD 1.57 lower (1.68 lower to 1.46 lower)	Moderate	IMPORTANT
TG	3	Randomized trials	Serious	Serious^a^	Not serious	Not serious	None	148	148	–	MD 0.16 lower (0.25 lower to 0.06 lower)	Low^a^	IMPORTANT
TC	3	Randomized trials	Serious	Not serious	Not serious	Not serious	None	148	148	–	MD 0.72 lower (0.81 lower to 0.63 lower)	Moderate	IMPORTANT
LDL-c	3	Randomized trials	Serious	Serious^a^	Not serious	Not serious	None	148	148	–	MD 0.2 lower (0.32 lower to 0.08 lower)	Low^a^	IMPORTANT
HDL-c	3	Randomized trials	Serious	Serious^a^	Not serious	Not serious	None	148	148	–	MD 0.11 higher (0.03 higher to 0.18 higher)	Low^a^	IMPORTANT

^a^ 50%< I^2^<75%.

^b^ I^2^> 75%.

### 3.2 *In silico* analysis results

**Figure 7:**
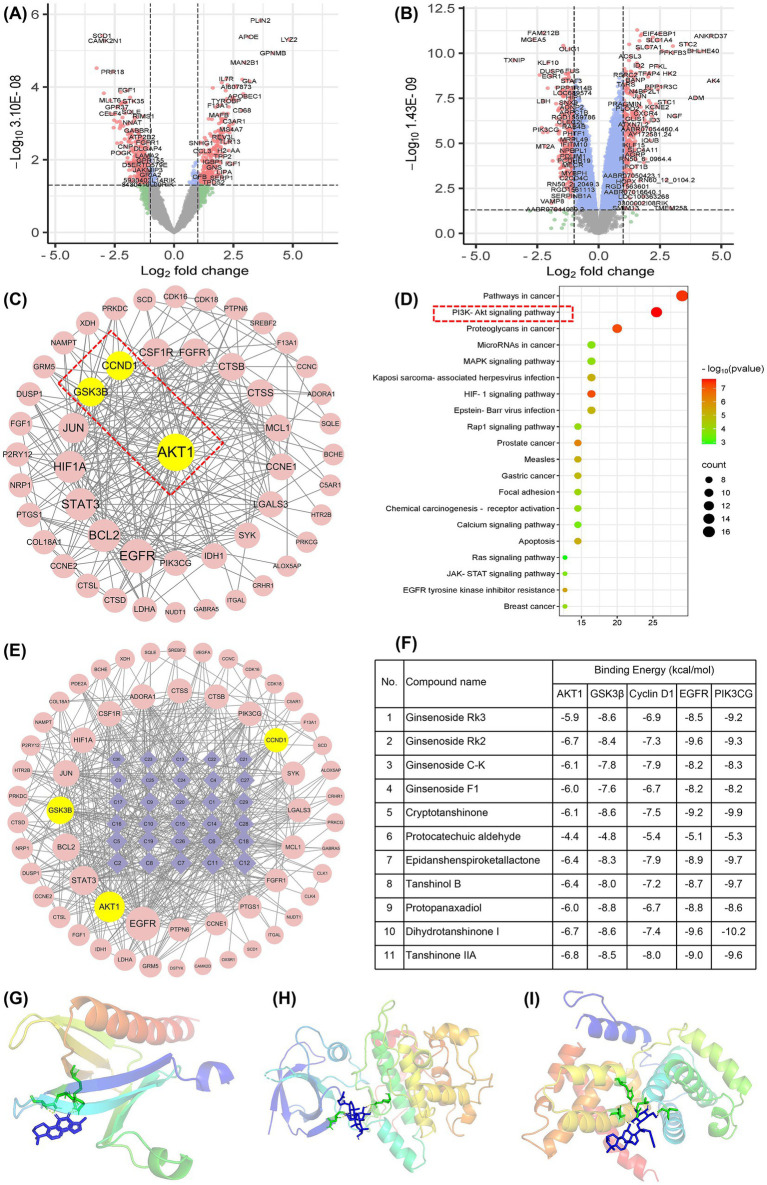
*In silico* analysis to explore the potential pharmacological mechanism of FDT in treating ischemic stroke. **(A)** Volcano map of DEGs in the GSE53737 dataset. **(B)** Volcano plot of DEGs in the GSE114609 dataset. **(C)** PPI network of intersection targets. **(D)** KEGG pathway enrichment analysis. **(E)** Component-target network. **(F)** The binding energy of the core active components and key targets. **(G)** Molecular docking of Tanshinone IIA and AKT. **(H)** Molecular docking of ginsenoside Rk3 and GSK3β. **(I)** Molecular docking of ginsenoside C-K and Cyclin D1.

Based on the high-throughput sequencing dataset combined with DESeq2 software, we identified 622 DEGs related to OPC injury in ischemic stroke from the GSE53737 dataset, including 297 down-regulated genes and 325 up-regulated genes (Figure 7A). Similarly, a total of 294 down-regulated genes and 309 up-regulated genes were screened out from the GSE114609 dataset (Figure 7B). Meanwhile, using the Swiss Target Prediction database, we obtained 817 drug target genes on the basis of the blood-entering components of FDT identified in previous studies (27). By intersecting these drug target genes with the DEGs of GSE53737 and GSE114609 datasets, 56 potential targets were obtained for FDT to regulate OPCs in the intervention of ischemic stroke.

For the PPI network, the above-mentioned 56 intersection target genes were introduced into the STRING database, and the TSV format files were downloaded. The TSV file was further imported into the Cytoscape software to depict the PPI network (Figure 7C). The importance of nodes was evaluated by degree and BC values to screen the core action targets of drug components. Obviously, AKT is a core target of FDT in treating ischemic stroke. Subsequently, we adopted the DAVID database to conduct GO function and KEGG pathway enrichment analysis on the intersection targets (Figure 7D). The above analysis indicates that the potential mechanism of FDT regulating OPCs in treating ischemic stroke mainly involves the AKT/GSK3β/Cyclin D1 pathway.

To evaluate the interaction between drug components and potential targets, we simultaneously imported 30 drug components and 56 potential targets into the Cytoscape software and outlined the component-target network (Figure 7E). The drug active ingredients from large to small based on the degree value, and the top-ranked potential active components were ginsenoside Rk3, Rk2, C-K, F1, cryptotanshinone, protocatechuic aldehyde, epidanshenspiroketallactone, tanshinol B, protopanaxadiol, dihydrotanshinone I, tanshinone IIA, etc. Furthermore, we conducted molecular docking between these core pharmacological components and the AKT/GSK3β/Cyclin D1 pathway, as well as its upstream targets PIK3CG and EGFR. The crystal structures of these target proteins were acquired from the PDB database, with the PDB ID for AKT1, GSK3β, Cyclin D1, EGFR, and PIK3CG being 2uzu, 4j1r, 2w96, 7aei, and 7jwe, respectively. It was found that the binding energies were all less than −5 kcal/mol (Figures 7F–I), showing a strong binding ability. These results suggested that FDT may regulate OPCs to treat ischemic stroke through the AKT/GSK3β/Cyclin D1 pathway.

## 4 Discussion

The incidence of ischemic stroke is increasing worldwide, and the disease population shows a younger trend. Ischemic stroke causes serious distress to patients and their families, and also takes up a great deal of medical resources. Therefore, it is urgent to obtain safe and therapeutic interventions to enable patients to achieve maximum recovery from the disease. TCM formula has a long history of treating stroke (38, 39). According to the Chinese National Stroke Database, 70% patients of ischemic stroke have been treated with TCM medicine, and the proportion of new stroke patients using TCM medicine is as high as 83.1%. Most of these TCM medicines aim to promote blood circulation and eliminate thrombus (40, 41). In fact, thrombus is the primary pathological mechanism of ischemic stroke. FDT is a modern TCM formula made of Dan-Shen, San-Qi, and Bing-Pian, and it has been demonstrated to be effective in improving blood circulation and eliminating thrombus. At present, FDT has been included in several clinical guidelines for the intervention of cardiovascular and cerebrovascular diseases (13). Although FDT has shown certain efficacy in the intervention of ischemic stroke, meta-analysis based summary and evaluation have not been conducted yet.

In this work, we systematically assessed the efficacy of FDT for the intervention of ischemic stroke by meta-analysis. 29 clinical trials with a total of 1,634 participants were involved. FDT was found to be an effective drug for improving neurologic functions, hemorheology indexes, inflammatory markers, and blood lipid indicators. The meta-analysis revealed that FDT-combined usual care (UC) significantly improved treatment efficiency compared to UC alone. A significant reduction in NIHSS score was observed, either in FDT combined intervention trials or FDT individual intervention trials. Notably, the mean difference value of the FDT individual intervention trials (MD = -6.05, 95%CI[−11.60, −0.51], *p* = 0.03) was lower than that of the FDT-combined intervention trials (MD = -3.52, 95%CI[−3.97, −3.08], *p* < 0.00001), possibly because of the significant heterogeneity of one study (33). After excluding this study, the mean difference values were comparable between the two groups. NIHSS is the most widely used neurologic deficit rating scale, providing a gold standard for stroke severity rating in clinical trials in vascular neurology (42). Meta-analysis results show that FDT could effectively promote the recovery of neurological function.

Generally speaking, thrombosis is the vital reason of ischemic stroke. Hemorheology mainly characterizes the status of blood circulation in vessels by determining the fluidity, deformability, and aggregation of whole blood, plasma, and blood tangible components (erythrocytes, leukocytes, and platelets), so as to providing objective basis for monitoring blood flow characteristics and evaluating therapeutic effects (43). In this work, the efficacy of FDT therapy on hemorheology in ischemic stroke patients was evaluated with four hemorheology indexes, including WBV-h, WBV-l, plasma viscosity, and HCT. The meta-analysis results revealed that either FDT combined with or without usual care could significantly lower the level of hemorheology indexes. Notably, the mean difference values of four hemorheology indexes in the FDT-combined intervention group were much lower than those in the FDT-individual intervention group, suggesting that FDT may contribute less to improving hemorheology.

In terms of inflammatory markers, two randomized controlled trials (RCTs) (31, 32) reported the level of TNF-*α*, IL-6, and hs-CRP in patients treated with FDT combined with usual care. Inflammation is one of the key components of the cascade of brain cell death in ischemic stroke patients, involving immune cells, such as microglia in the brain and peripheral white blood cells (such as T cells, neutrophils, monocytes, etc.) (44). After the onset of ischemic stroke, activated microglia release a mass of inflammatory mediators, such as chemokines and cytokines (e.g., TNF-α and IL-6) (45). In addition, inflammation will recruit a series of peripheral white blood cells, such as T cells, neutrophils, and monocytes, into the brain, which subsequently become important mediators of acute neuroinflammation during ischemic stroke through continuous release of IL-6 and TNF-α (46). The inflammatory cascade eventually leads to a series of cerebral damages, such as the disorder of the blood–brain barrier, neuron damage, and vascular aging. Meta-analysis results show that FDT can effectively suppress the spike of TNF-α, IL-6, and hs-CRP in ischemic stroke patients so as to reduce brain tissue injuries.

Hyperlipidemia is an independent risk factor for stroke (47, 48). Excess lipids can deposit under the lining of blood vessels, causing atherosclerotic plaques, narrowing the lumen of the artery, blocking blood flow to the appropriate area, which can lead to ischemic stroke. A meta-analysis of the lipid-lowering drug statins revealed that stroke risk was reduced by 21.1% if LDL-c was reduced by 1 mmol/L (49). Therefore, reducing blood lipid levels is necessary for the intervention of ischemic stroke. Based on 14 clinical studies (28, 34, 35, 50–60), the effects of FDT therapy on the level of TG, TC, LDL-c, and HDL-c were evaluated. The meta-analysis results reveal that FDT therapy can notably reduce the levels of TG, TC, and LDL and promote HDL in ischemic stroke patients. Based on the comparison of mean difference values, the lipid-lowering effect of FDT individual intervention is no less than that of FDT combined with usual care.

Nevertheless, there exist several flaws in the current meta-analysis. First, a few RCTs were retrieved and finally incorporated into the meta-analysis. Most included trials were of low quality in methodological design, with unclear descriptions of randomized methods, blind methods, and concealed assignments, resulting in a certain impact on the reliability of evidence. Second, there was heterogeneity in patient population, composition of usual care, time from onset to enrollment, and duration of drug treatment in different studies, which may lead to bias. Third, there was a lack of follow-up trials of long-term, making it difficult to evaluate the long-term effects of FDT intervention. Hence, more high-quality, well-designed, long-term RCTs are urgently needed to determine the therapeutic efficacy of FDT on ischemic stroke.

Remyelination mediated by oligodendrocyte precursor cells (OPCs) is the key step in restoring neurological deficits in ischemic stroke (25, 26). Herein, we explored the potential pharmacological mechanism of FDT in treating ischemic stroke from the perspective of regulating OPCs on the basis of GEO datasets, network pharmacology, and molecular docking. A total of 1,225 DEGs and 817 drug target genes were screened, giving rise to the intersection of 56 potential targets for the regulation of OPCs by FDT in the treatment of ischemic stroke. As shown in the PPI network (Figure 7C), AKT plays a core role in the regulation of OPCs by FDT. Further, the KEGG pathway enrichment analysis (Figure 7D) suggested the core role of AKT/GSK3β/Cyclin D1 for FDT, regulating OPCs in treating ischemic stroke.

AKT, a central component of the PI3K/AKT signaling pathway, plays a crucial role in regulating various physiological processes, such as cell survival, proliferation, and metabolism (61). As the AKT pathway itself exerts anti-inflammatory effects, its activation may indirectly account for the observed improvement in inflammatory markers (62). Meanwhile, a series of studies have revealed that the activation, migration, proliferation, and differentiation of oligodendrocyte precursor cells (OPCs) are closely associated with the AKT signaling pathway. For instance, oligodendrocyte-derived sirtuin 2 can target the AKT/GSK-3β pathway to regulate neuroplasticity (63). The AKT/GSK3β pathway could promote OPC differentiation, maturation, and myelin repair by modulating cyclin-dependent kinase 5 (64). This direct regulatory role of AKT on OPCs links it directly to neurological functional recovery, as reflected in improvements in NIHSS scores. Moreover, remyelination is a progressive process. The signaling pathways controlling OPC differentiation and proliferation are mutually exclusive. OPCs must exit the cell cycle to initiate differentiation. This transition critically involves the cell cycle regulator Cyclin D1, a key protein whose expression and function are intimately tied to OPC proliferation, differentiation, and myelination (65). Therefore, targeting the AKT/GSK3β/Cyclin D1 signaling pathway to modulate OPC differentiation represents a potentially viable strategy for promoting remyelination and rehabilitation following ischemic stroke.

In recent years, the medicinal potential of natural products and traditional Chinese medicine in promoting remyelination has received increasing attention (66). For example, ginsenoside Rg_1_ was found to promote the proliferation of OPCs and restore the myelin sheath structure by intervening in the AKT/GSK3β pathway (67). To explore the primary pharmacodynamic components of FDT, a component-target network was depicted with Cytoscape software. Moreover, molecular docking was conducted between the top-ranked active components and potential key targets, i.e., AKT/GSK3β/Cyclin D1 pathway and its upstream targets PIK3CG and EGFR. The calculated binding energies were all less than −5 kcal/mol (Figure 7F), showing a strong binding ability. These results indicate that FDT can regulate OPCs by acting on multiple targets on the AKT/GSK3β/Cyclin D1 pathway. However, several issues should be noted. On the one side, the FDT-derived components included in this study were blood-entering compounds. Due to the obstruction of the blood–brain barrier, the compounds in the blood may not be able to enter the brain tissue. Besides, the components included in this study mainly originated from Dan-Shen and San-Qi, and did not involve Bing-Pian. Therefore, the comprehensive identification of drug components in the brain is an important part of subsequent research. On the other hand, the multi-target mode of action also makes the mechanism verification more complex. Specific inhibitors and target gene knockout are effective strategies, which means more experiments and costs. In other words, further confirmation experiments are indispensable.

## 5 Conclusion

The current systematic review took neurologic functional scale, hemorheology indexes, inflammatory markers, and blood–lipid indicators into serious consideration so as to determine the therapeutic effects of FDT intervention on ischemic stroke for the first time. Meta-analysis results revealed that FDT therapy could effectively enhance neurological recovery, promote blood circulation, inhibit the inflammatory cascade, and lower the blood lipid level in patients with ischemic stroke. Further network pharmacology and molecular docking analysis emphasized the potentially important role of the AKT/GSK3β/Cyclin D1 pathway for FDT regulating OPCs in treating ischemic stroke. However, there are several limitations in this work. On the one hand, high-quality RCTs of FDT in intervening ischemic stroke are still scarce. Most reported clinical trials have flaws in methodological design, follow-up management, and heterogeneity assessment. More long-term follow-up RCTs of high quality are essential to further demonstrate the efficacy and safety. On the other hand, the ambiguity of FDT components entering the brain restricts the clarification of the pharmacodynamic substances and pharmacological mechanisms. Although in-depth network pharmacology and molecular docking analysis were carried out, further experimental studies are indispensable to validate the effects of FDT in regulating OPCs of ischemic stroke.

## Data Availability

The original contributions presented in the study are included in the article/Supplementary material, and further inquiries can be directed to the corresponding authors.
