# Supplementary material for: The efficacy and potential pharmacological mechanism of Fufang Danshen Tablet in promoting the rehabilitation of ischemic stroke: a meta-analysis and in silico study
Source: Front Neurol. 2026 Feb 12;17:1724630. doi: 10.3389/fneur.2026.1724630 (PMC12935622; doi:10.3389/fneur.2026.1724630)
Supplement: Supplementary file 1 [file Data_Sheet_1.PDF]

# *Supplementary Material*

**Table of Contents**


Supplementary Figure 1. Forest plot for the meta-analysis of WBV-h. (A) FDT combined intervention studies. (B) FDT individual intervention studies.

Supplementary Figure 2. Forest plot for the meta-analysis of WBV-l. (A) FDT combined intervention studies. (B) FDT individual intervention studies.

Supplementary Figure 3. Forest plot for the meta-analysis of plasma viscosity. (A) FDT combined intervention studies. (B) FDT individual intervention studies.

Supplementary Figure 4. Forest plot for the meta-analysis of HCT value. (A) FDT combined intervention studies. (B) FDT individual intervention studies.

Supplementary Figure 5. Forest plot for the meta-analysis of IL-6 (A), TNF-α (B), and hs-CRP (C) level.

Supplementary Figure 6. Forest plot for the meta-analysis of TG level. (A) FDT combined intervention studies. (B) FDT individual intervention studies.

Supplementary Figure 7. Forest plot for the meta-analysis of TC level. (A) FDT combined intervention studies. (B) FDT individual intervention studies.

Supplementary Figure 8. Forest plot for the meta-analysis of LDL-c level.

Supplementary Figure 9. Forest plot for the meta-analysis of HDL-c level. (A) FDT combined intervention studies. (B) FDT individual intervention studies.

Supplementary Figure 10. The Egger's test of whole blood viscosity at high shear rate.

Supplementary Figure 11. The Egger's test of whole blood viscosity at low shear rate.

Supplementary Figure 12. The Egger's test of plasma viscosity.

Supplementary Figure 13. The Egger's test of HCT.

Supplementary Figure 14. The Egger's test of TG.

Supplementary Figure 15. The Egger's test of TC.

Supplementary Figure 16. The Egger's test of HDL-c.

Supplementary Table 1. Chemical information of 30 components identified in rat serum collected after oral administration of FDT.

Supplementary Table 2. GRADE evidence profiles of FDT individual intervention trails.

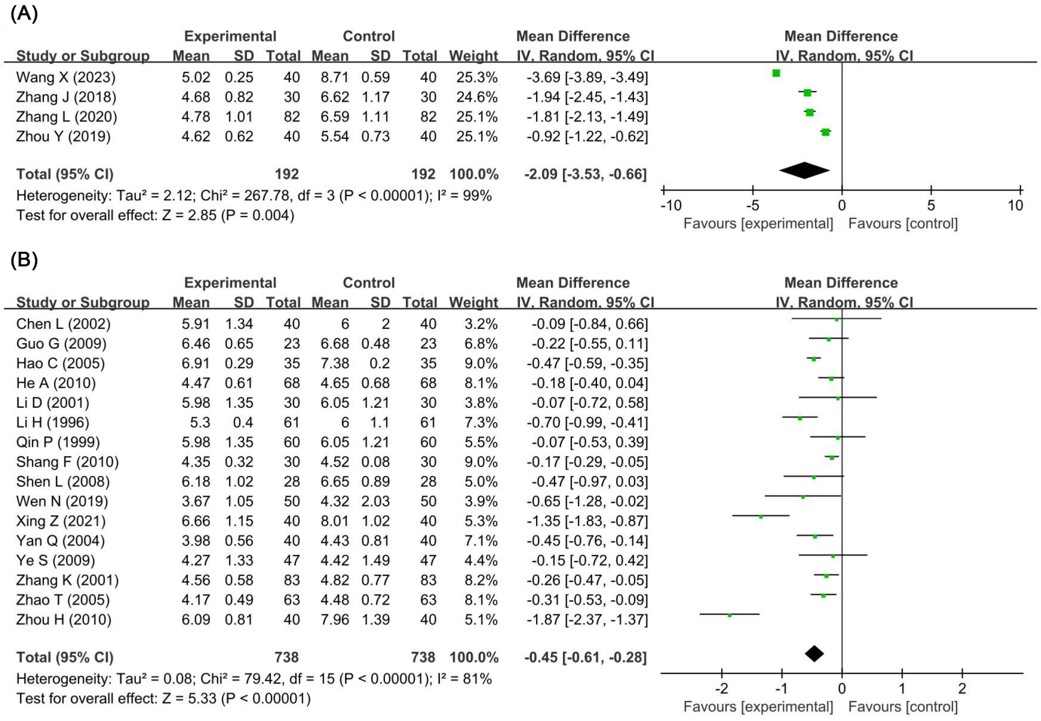


**Supplementary Figure 1.** Forest plot for the meta-analysis of WBV-h. (A) FDT combined intervention studies. (B) FDT individual intervention studies.

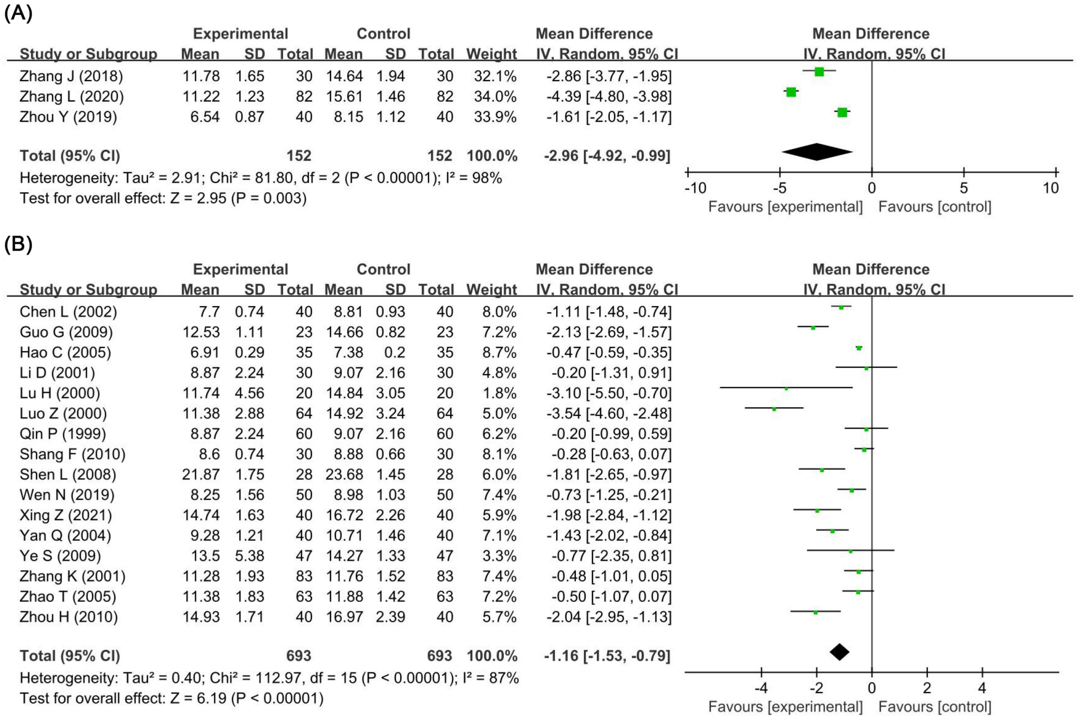


**Supplementary Figure 2.** Forest plot for the meta-analysis of WBV-l. (A) FDT combined intervention studies. (B) FDT individual intervention studies.

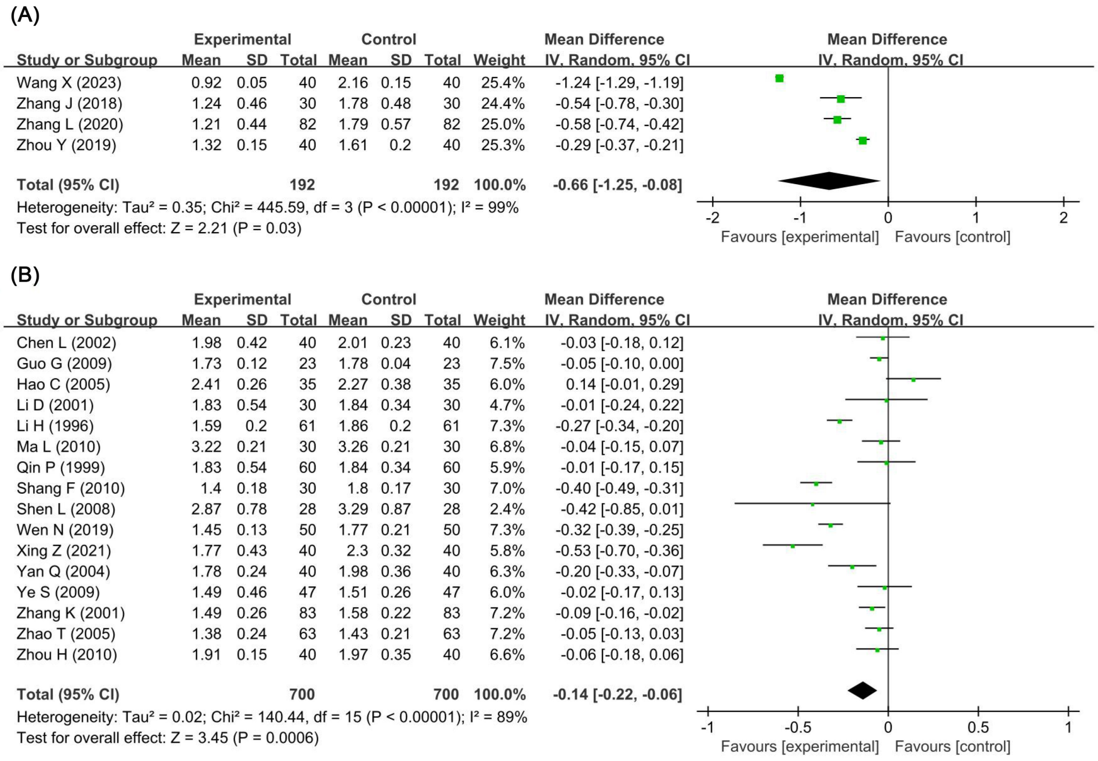


**Supplementary Figure 3.** Forest plot for the meta-analysis of plasma viscosity. (A) FDT combined intervention studies. (B) FDT individual intervention studies.

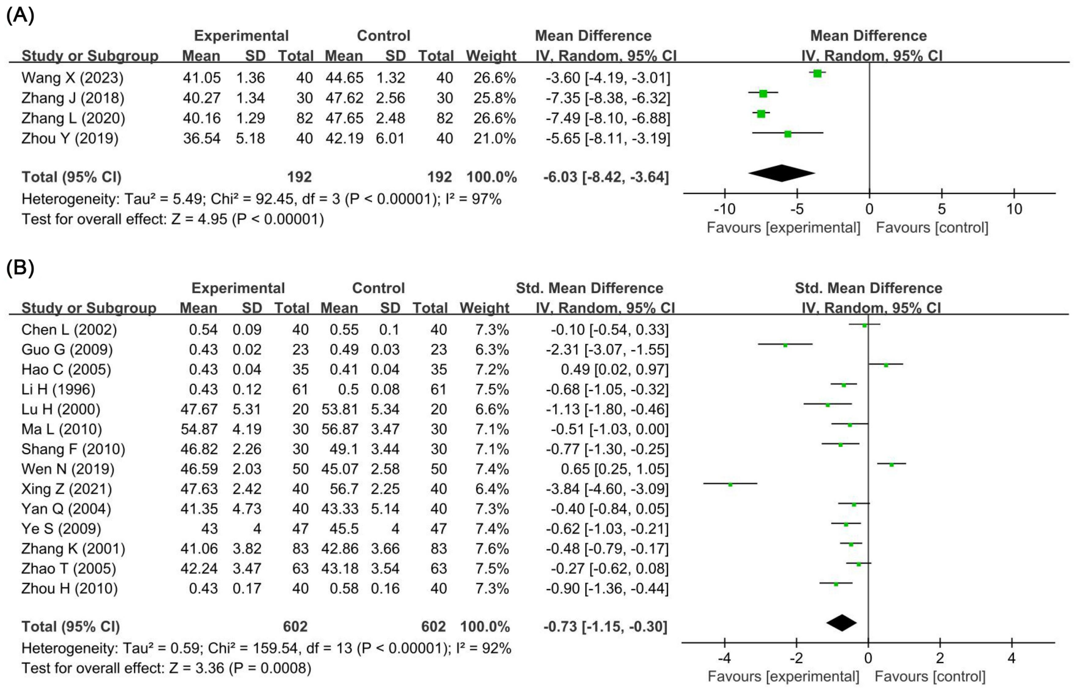


**Supplementary Figure 4.** Forest plot for the meta-analysis of HCT value. (A) FDT combined intervention studies. (B) FDT individual intervention studies.

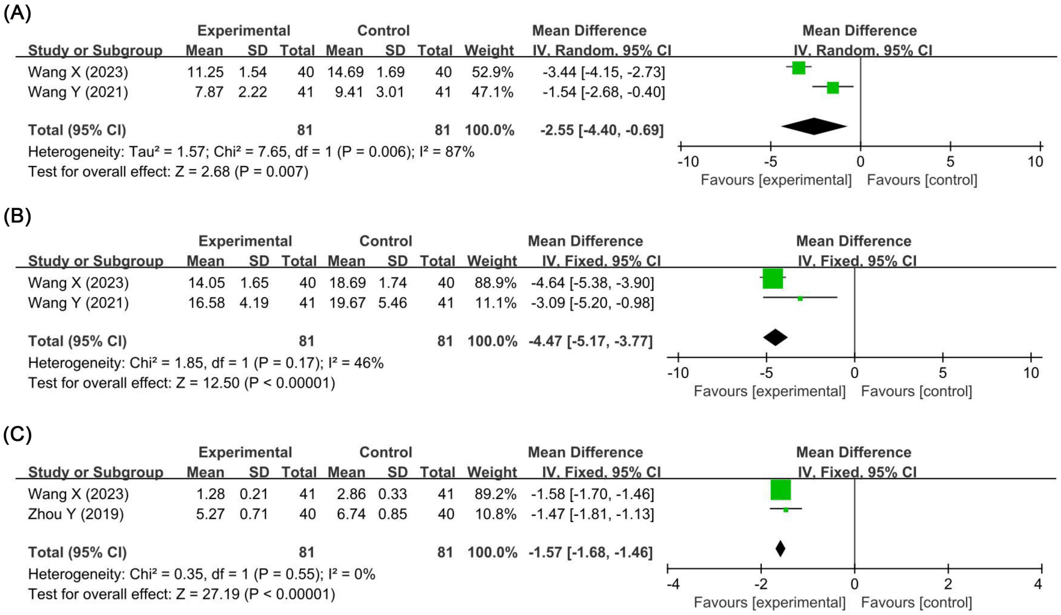


**Supplementary Figure 5.** Forest plot for the meta-analysis of IL-6 (A), TNF-α (B), and hs-CRP (C) level.

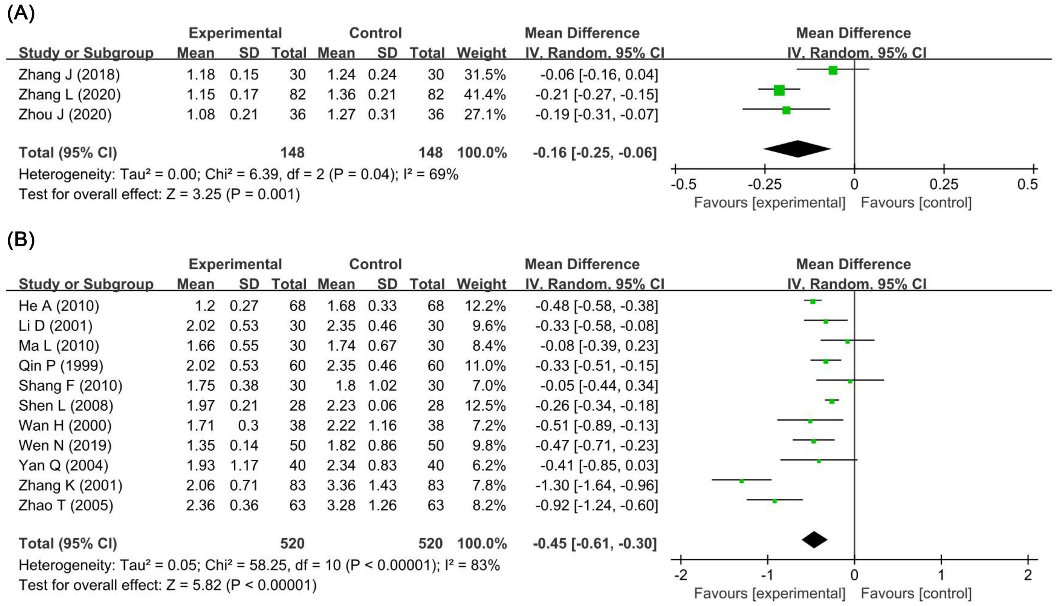


**Supplementary Figure 6.** Forest plot for the meta-analysis of TG level. (A) FDT combined intervention studies. (B) FDT individual intervention studies.

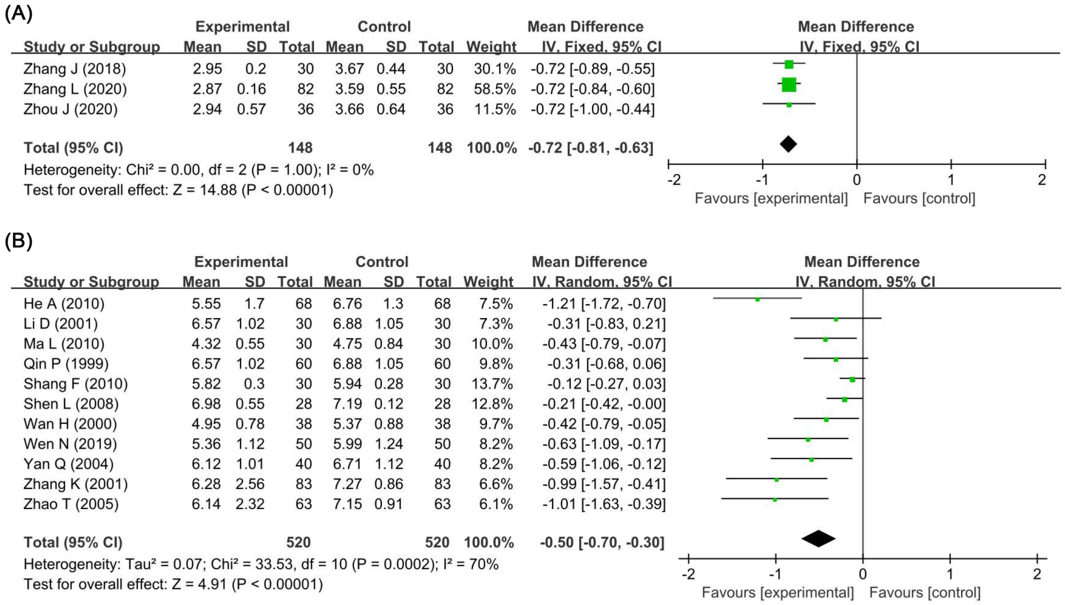


**Supplementary Figure 7.** Forest plot for the meta-analysis of TC level. (A) FDT combined intervention studies. (B) FDT individual intervention studies.

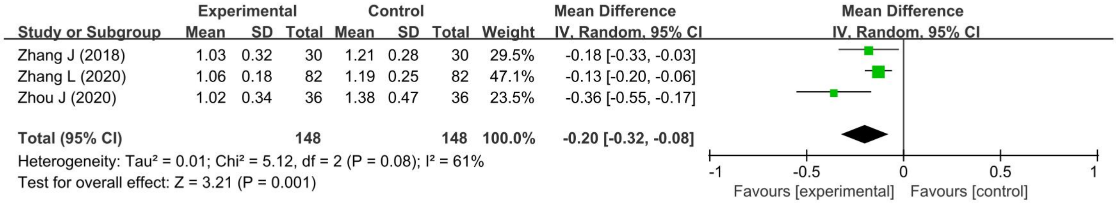


**Supplementary Figure 8.** Forest plot for the meta-analysis of LDL-c level.

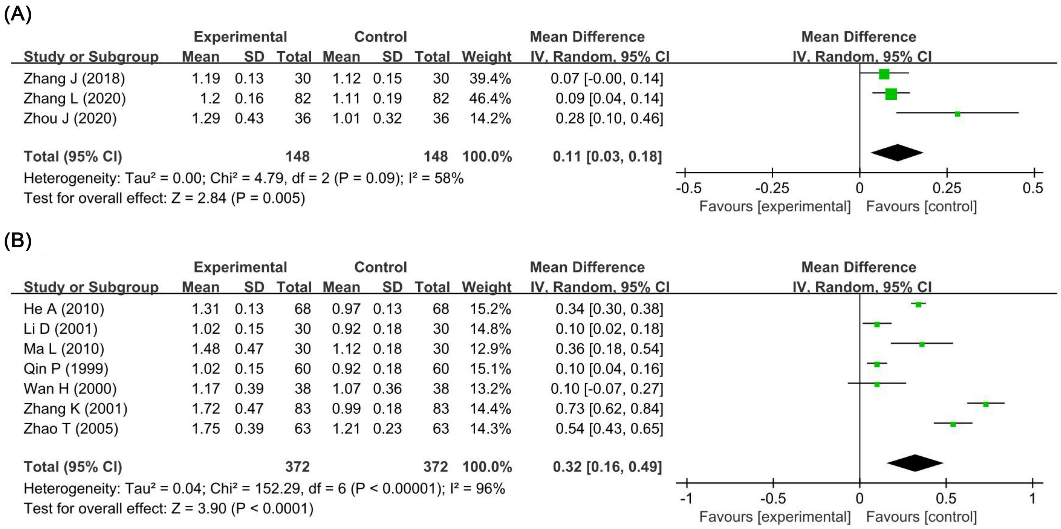


**Supplementary Figure 9.** Forest plot for the meta-analysis of HDL-c level. (A) FDT combined intervention studies. (B) FDT individual intervention studies.

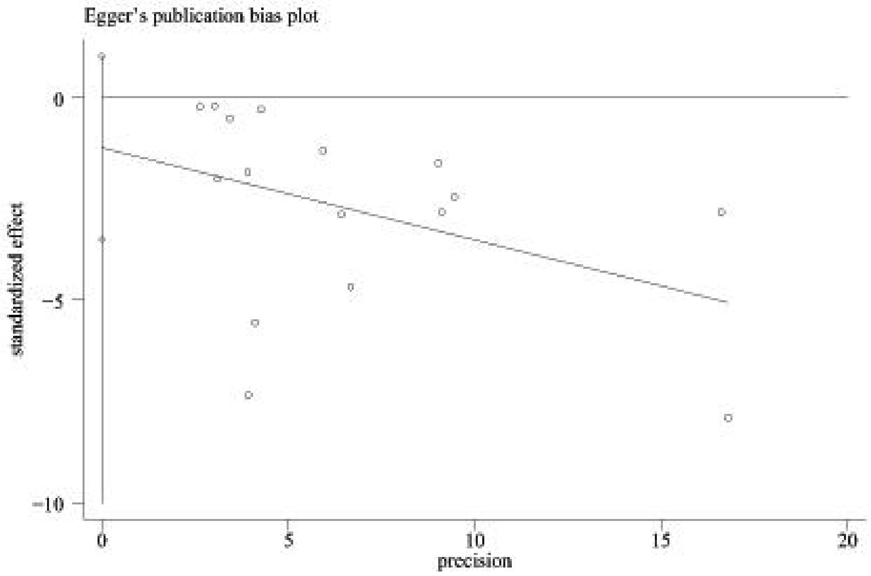


Egger's test

| Std_Eff | Coefficient | Std. err. | t | P>\|t\| | [95% conf. interval] | |
|---|---|---|---|---|---|---|
| slope | -.2271407 | .1308811 | -1.74 | 0.105 | -.5078527 | .0535713 |
| bias | -1.242427 | 1.053131 | -1.18 | 0.258 | -3.501168 | 1.016314 |

**Supplementary Figure 10.** The Egger's test of whole blood viscosity at high shear rate.

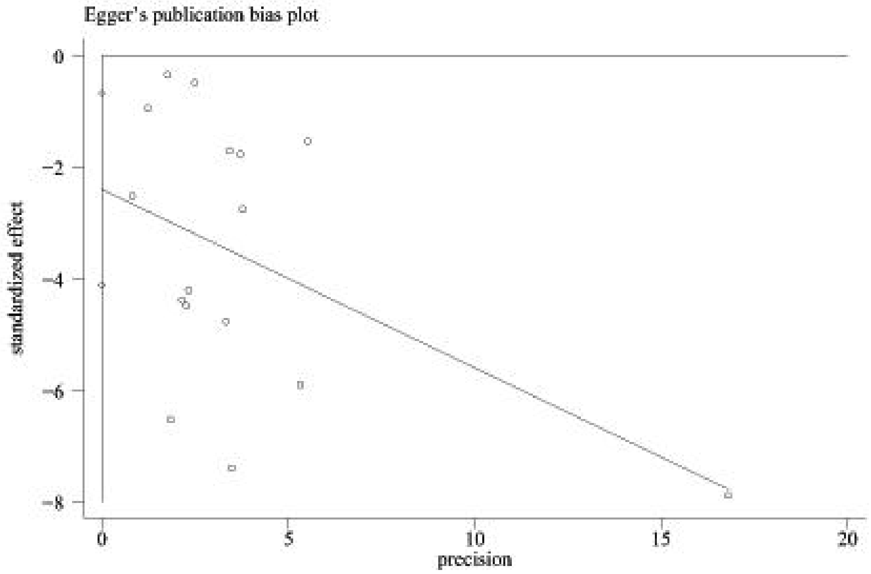


Egger's test

| Std_Eff | Coefficient | Std. err. | t | P>\|t\| | [95% conf. interval] | |
|---|---|---|---|---|---|---|
| slope | -.320024 | .1542054 | -2.08 | 0.057 | -.6507616 | .0107136 |
| bias | -2.402993 | .8030949 | -2.99 | 0.010 | -4.12546 | -.6805254 |

**Supplementary Figure 11.** The Egger's test of whole blood viscosity at low shear rate.

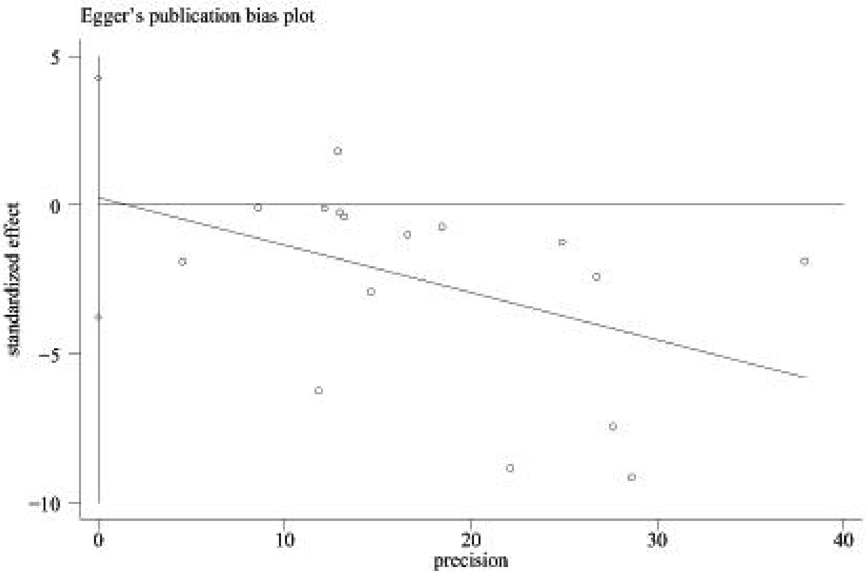


Egger's test

| Std_Eff | Coefficient | Std. err. | t | P>\|t\| | [95% conf. interval] | |
|---|---|---|---|---|---|---|
| slope | -.1594567 | .0924495 | -1.72 | 0.107 | -.3577412 | .0388277 |
| bias | .246012 | 1.872297 | 0.13 | 0.897 | -3.769665 | 4.261689 |

**Supplementary Figure 12.** The Egger's test of plasma viscosity.

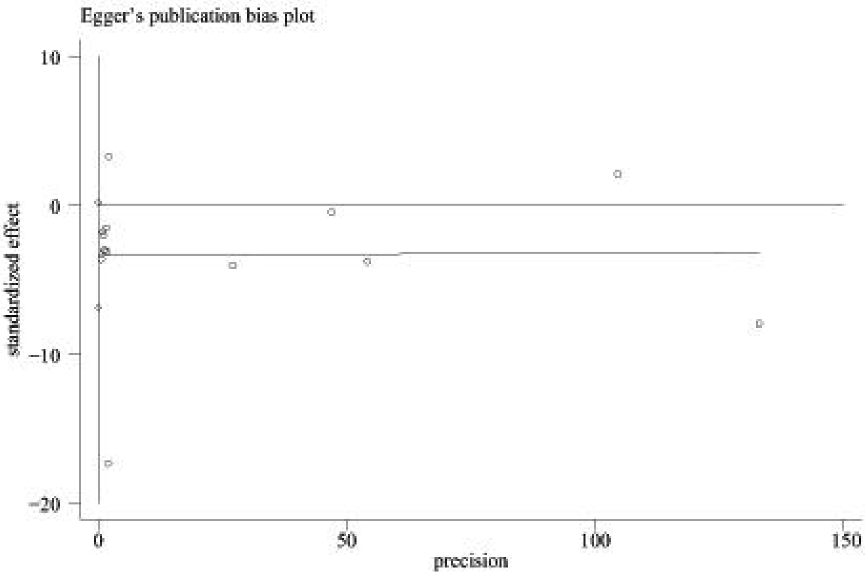


Egger's test

| Std_Eff | Coefficient | Std. err. | t | P>\|t\| | [95% conf. interval] | |
|---|---|---|---|---|---|---|
| slope | .001095 | .0325041 | 0.03 | 0.974 | -.0697252 | .0719153 |
| bias | -3.345372 | 1.614153 | -2.07 | 0.060 | -6.862309 | .1715642 |

**Supplementary Figure 13.** The Egger's test of HCT.

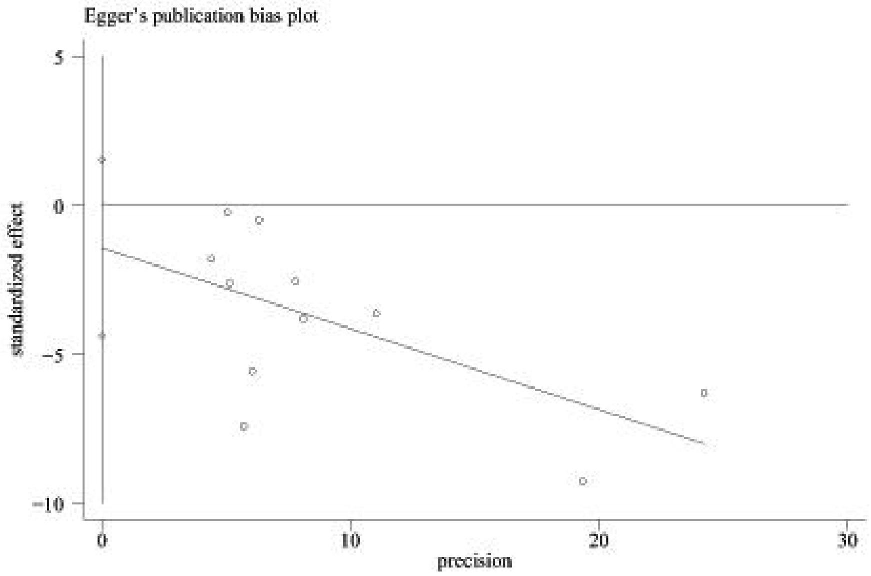


Egger's test

| Std_Eff | Coefficient | Std. err. | t | P>\|t\| | [95% conf. | interval] |
|---|---|---|---|---|---|---|
| slope | -.2715599 | .1163253 | -2.33 | 0.044 | -.5347061 | -.0084138 |
| bias | -1.433704 | 1.307651 | -1.10 | 0.301 | -4.391817 | 1.524409 |

**Supplementary Figure 14.** The Egger's test of TG.

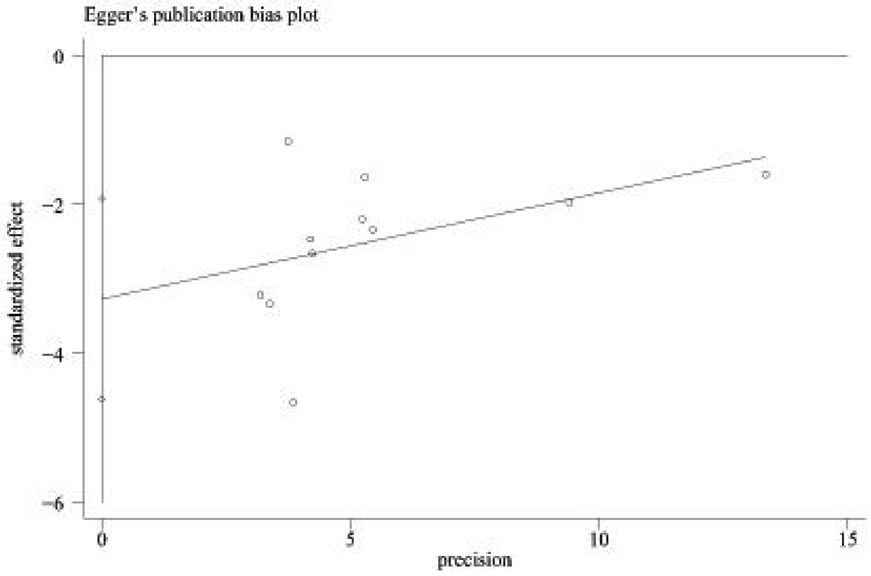


Egger's test

| Std_Eff | Coefficient | Std. err. | t | P>\|t\| | [95% conf. | interval] |
|---|---|---|---|---|---|---|
| slope | .1424109 | .0945232 | 1.51 | 0.166 | -.0714155 | .3562372 |
| bias | -3.274036 | .5961902 | -5.49 | 0.000 | -4.622712 | -1.92536 |

**Supplementary Figure 15.** The Egger's test of TC.

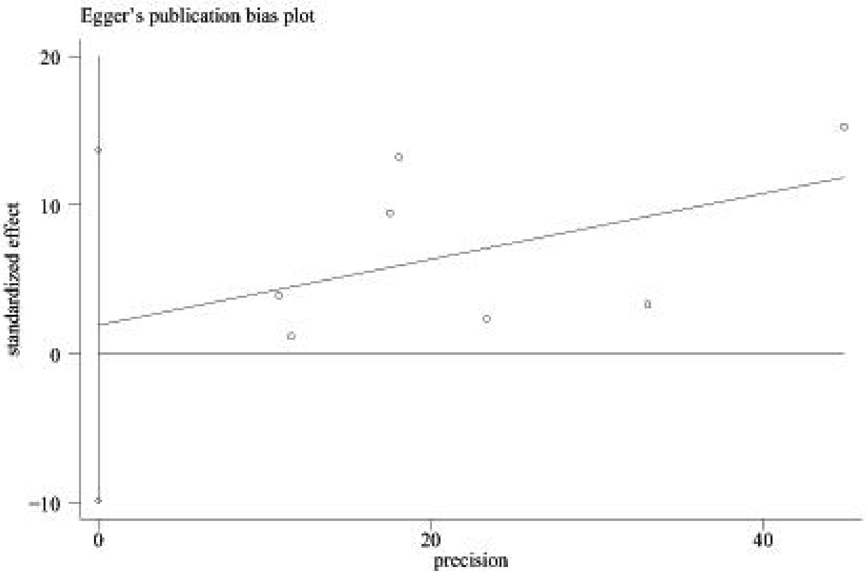


Egger's test

| Std_Eff | Coefficient | Std. err. | t | P>\|t\| | [95% conf. interval] | |
|---|---|---|---|---|---|---|
| slope | .2213615 | .1799645 | 1.23 | 0.273 | -.241252 | .683975 |
| bias | 1.909237 | 4.582917 | 0.42 | 0.694 | -9.871526 | 13.69 |

**Supplementary Figure 16.** The Egger's test of HDL-c.

**Supplementary Table 1.** Chemical information of 30 components identified in rat serum collected after oral administration of FDT

| No. | Compound name | Formula | PubChem CID | SMILES number |
|---|---|---|---|---|
| 1 | Caffeic acid | $C_9H_8O_4$ | 689043 | C1=CC(=C(C=C1/C=C/C(=O)O)O)O |
| 2 | Cryptotanshinone | $C_{19}H_{20}O_3$ | 160254 | C[C@H]1COC2=C1C(=O)C(=O)C3=C2C=CC4=C3CCCC4(C)C |
| 3 | Danshensu | $C_9H_{10}O_5$ | 11600642 | C1=CC(=C(C=C1C[C@H](C(=O)O)O)O)O |
| 4 | Danshenxinkun A | $C_{18}H_{16}O_4$ | 149138 | CC1=C2C=CC3=C(C2=CC=C1)C(=O)C(=O)C(=C3O)C(C)CO |
| 5 | Dihydrotanshinone I | $C_{18}H_{14}O_3$ | 11425923 | C[C@H]1COC2=C1C(=O)C(=O)C3=C2C=CC4=C(C=CC=C43)C |
| 6 | Epidanshenspiroketallactone | $C_{17}H_{16}O_3$ | 102004791 | C[C@H]1C[C@@]2(C3=C(C4=CC=CC(=C4C=C3)C)C(=O)O2)OC1 |
| 7 | Ginsenoside C-K | $C_{36}H_{62}O_8$ | 9852086 | CC(=CCC[C@@](C)([C@H]1CC[C@@]2([C@@H]1[C@@H](C[C@H]3[C@]2(CC[C@@H]4[C@@]3(CC[C@@H](C4(C)C)O)C)C)O)C)O[C@H]5[C@@H]([C@H]([C@@H]([C@H](O5)CO)O)O)O)C |
| 8 | Ginsenoside F1 | $C_{36}H_{62}O_9$ | 9809542 | CC(=CCC[C@@](C)([C@H]1CC[C@@]2([C@@H]1[C@@H](C[C@H]3[C@]2(C[C@@H]([C@@H]4[C@@]3(CC[C@@H](C4(C)C)O)C)O)C)O)C)O[C@H]5[C@@H]([C@H]([C@@H]([C@H](O5)CO)O)O)O)C |
| 9 | Ginsenoside F2 | $C_{42}H_{72}O_{13}$ | 9918692 | CC(=CCC[C@@](C)([C@H]1CC[C@@]2([C@@H]1[C@@H](C[C@H]3[C@]2(CC[C@@H]4[C@@]3(CC[C@@H](C4(C)C)O[C@H]5[C@@H]([C@H]([C@@H]([C@H](O5)CO)O)O)O)C)C)O)C)O[C@H]6[C@@H]([C@H]([C@@H]([C@H](O6)CO)O)O)O)C |
| 10 | Ginsenoside Rg1 | $C_{42}H_{72}O_{14}$ | 441923 | CC(=CCC[C@@](C)([C@H]1CC[C@@]2([C@@H]1[C@@H](C[C@H]3[C@]2(C[C@@H]([C@@H]4[C@@]3(CC[C@@H](C4(C)C)O)C)O[C@H]5[C@@H]([C@H]([C@@H]([C@H](O5)CO)O)O)O)C)O)C)O[C@H]6[C@@H]([C@H]([C@@H]([C@H](O6)CO)O)O)O)C |
| 11 | Ginsenoside Rk2 | $C_{36}H_{60}O_7$ | 90472238 | CC(=CCCC(=C)[C@H]1CC[C@@]2([C@@H]1[C@@H](C[C@H]3[C@]2(CC[C@@H]4[C@@]3(CC[C@@H](C4(C)C)O[C@H]5[C@@H]([C@H]([C@@H]([C@H](O5)CO)O)O)O)C)C)O)C)C |
| 12 | Ginsenoside Rk3 | $C_{36}H_{60}O_8$ | 75412555 | CC(=CCCC(=C)[C@H]1CC[C@@]2([C@@H]1[C@@H](C[C@H]3[C@]2(C[C@@H]([C@@H]4[C@@]3(CC[C@@H](C4(C)C)O)C)O[C@H]5[C@@H]([C@H]([C@@H]([C@H](O5)CO)O)O)O)C)O)C)C |
| 13 | Lithospermic acid | $C_{27}H_{22}O_{12}$ | 6441498 | C1=CC(=C(C=C1C[C@H](C(=O)O)OC(=O)/C=C/C2=C3[C@@H]([C@H](OC3=C(C=C2)O)C4=CC(=C(C=C4)O)O)C(=O)O)O)O |
| 14 | Methyltanshinonate | $C_{20}H_{18}O_5$ | 624381 | CC1=COC2=C1C(=O)C(=O)C3=C2C=CC4=C3CCCC4(C)C(=O)OC |
| 15 | Neocryptotanshinone | $C_{19}H_{22}O_4$ | 389888 | C[C@@H](CO)C1=C(C2=C(C3=C(C=C2)C(CCC3)(C)C)C(=O)C1=O)O |
| 16 | Nortanshinone | $C_{17}H_{12}O_4$ | 10062187 | CC1=COC2=C1C(=O)C(=O)C3=C2C=CC4=C3CCCC4=O |
| 17 | Protocatechuic acid | $C_7H_6O_4$ | 72 | C1=CC(=C(C=C1C(=O)O)O)O |
| 18 | Protocatechuic aldehyde | $C_7H_6O_3$ | 8768 | C1=CC(=C(C=C1C=O)O)O |
| 19 | Protopanaxadiol | $C_{30}H_{52}O_3$ | 9920281 | CC(=CCC[C@](C)([C@H]1CC[C@@]2([C@@H]1[C@@H](C[C@H]3[C@]2(CC[C@@H]4[C@@]3(CC[C@@H](C4(C)C)O)C)C)O)C)O)C |
| 20 | Protopanaxatriol | $C_{30}H_{52}O_4$ | 9847853 | CC(=CCC[C@](C)([C@H]1CC[C@@]2([C@@H]1[C@@H](C[C@H]3[C@]2(C[C@@H]([C@@H]4[C@@]3(CC[C@@H](C4(C)C)O)C)O)C)O)C)O)C |
| 21 | Rosmarinic acid | $C_{18}H_{16}O_8$ | 5281792 | C1=CC(=C(C=C1C[C@H](C(=O)O)OC(=O)/C=C/C2=CC(=C(C=C2)O)O)O)O |
| 22 | Salvianolic acid A | $C_{26}H_{22}O_{10}$ | 5281793 | C1=CC(=C(C=C1C[C@H](C(=O)O)OC(=O)/C=C/C2=C(C(=C(C=C2)O)O)/C=C/C3=CC(=C(C=C3)O)O)O)O |
| 23 | Salvianolic acid B | $C_{36}H_{30}O_{16}$ | 6451084 | C1=CC(=C(C=C1C[C@H](C(=O)O)OC(=O)/C=C/C2=C3[C@@H]([C@H](OC3=C(C=C2)O)C4=CC(=C(C=C4)O)O)C(=O)O[C@H](CC5=CC(=C(C=C5)O)O)C(=O)O)O)O |
| 24 | Salvianolic acid C | $C_{26}H_{20}O_{10}$ | 13991590 | C1=CC(=C(C=C1C[C@H](C(=O)O)OC(=O)/C=C/C2=C3C=C(OC3=C(C=C2)O)C4=CC(=C(C=C4)O)O)O)O |
| 25 | Salvianolic acid D | $C_{20}H_{18}O_{10}$ | 75412558 | C1=CC(=C(C=C1C[C@H](C(=O)O)OC(=O)/C=C/C2=C(C(=C(C=C2)O)O)CC(=O)O)O)O |
| 26 | Tanshinol B | $C_{18}H_{16}O_4$ | 126071 | CC1=COC2=C1C(=O)C(=O)C3=C2C=CC4=C3CCCC4(C)O |
| 27 | Tanshinone I | $C_{18}H_{12}O_3$ | 114917 | CC1=C2C=CC3=C(C2=CC=C1)C(=O)C(=O)C4=C3OC=C4C |
| 28 | Tanshinone IIA | $C_{19}H_{18}O_3$ | 164676 | CC1=COC2=C1C(=O)C(=O)C3=C2C=CC4=C3CCCC4(C)C |
| 29 | Tanshinone IIB | $C_{19}H_{18}O_4$ | 9926694 | CC1=COC2=C1C(=O)C(=O)C3=C2C=CC4=C3CCC[C@]4(C)CO |
| 30 | Vanillactic acid | $C_{10}H_{12}O_5$ | 160637 | COC1=C(C=CC(=C1)CC(C(=O)O)O)O |

**Supplementary Table 2.** GRADE evidence profiles of FDT individual intervention trails.

| Outcomes | Certainty assessment | | | | | | | № of patients | | Effect | | Certainty | Importance |
|---|---|---|---|---|---|---|---|---|---|---|---|---|---|
| | Number of studies | Study design | Risk of bias | Inconsistency | Indirectness | Imprecision | Other considerations | Treatment with FDT combined usual care | Treatment with usual care | Relative (95% CI) | Absolute (95% CI) | | |
| **NIHSS** | 4 | non-randomized studies | serious | very serious[a] | not serious | not serious | none | 246 | 246 | - | MD **6.05 lower** (11.6 lower to 0.51 lower) | ⊕◯◯◯ Very low[a] | CRITICAL |
| **Whole blood viscosity at high shear rate** | 16 | non-randomized studies | serious | very serious[a] | not serious | not serious | none | 738 | 738 | - | MD **0.45 lower** (0.61 lower to 0.28 lower) | ⊕◯◯◯ Very low[a] | IMPORTANT |
| **Whole blood viscosity at low shear rate** | 16 | non-randomized studies | serious | very serious[a] | not serious | not serious | publication bias strongly suspected[b] | 693 | 693 | - | MD **1.16 lower** (1.53 lower to 0.79 lower) | ⊕◯◯◯ Very low[a,b] | IMPORTANT |
| **Plasma viscosity** | 16 | non-randomized studies | serious | very serious[a] | not serious | not serious | none | 700 | 700 | - | MD **0.14 lower** (0.22 lower to 0.06 lower) | ⊕◯◯◯ Very low[a] | IMPORTANT |
| **HCT** | 14 | non-randomized studies | serious | very serious[a] | not serious | not serious | none | 602 | 602 | - | SMD **0.73 lower** (1.15 lower to 0.3 lower) | ⊕◯◯◯ Very low[a] | IMPORTANT |
| **TG** | 11 | non-randomized studies | serious | very serious[a] | not serious | not serious | none | 520 | 520 | - | MD **0.45 lower** (0.61 lower to 0.3 lower) | ⊕◯◯◯ Very low[a] | IMPORTANT |
| **TC** | 11 | non-randomized studies | serious | serious[c] | not serious | not serious | publication bias strongly suspected[b] | 520 | 520 | - | MD **0.5 lower** (0.7 lower to 0.3 lower) | ⊕◯◯◯ Very low[b,c] | IMPORTANT |
| **HDL-c** | 7 | non-randomized studies | serious | very serious[a] | not serious | not serious | none | 372 | 372 | - | MD **0.32 higher** (0.16 higher to 0.49 higher) | ⊕◯◯◯ Very low[a] | IMPORTANT |

a. $I^2>75\%$. b. The *P*-value of Egger's test was less than 0.05. c. $50\%<I^2<75\%$.
